# Functional Dissection of the Chickpea (*Cicer arietinum L.*) Stay-Green Phenotype Associated with Molecular Variation at an Ortholog of Mendel’s I Gene for Cotyledon Color: Implications for Crop Production and Carotenoid Biofortification

**DOI:** 10.3390/ijms20225562

**Published:** 2019-11-07

**Authors:** Kaliamoorthy Sivasakthi, Edward Marques, Ng’andwe Kalungwana, Noelia Carrasquilla-Garcia, Peter L. Chang, Emily M. Bergmann, Erika Bueno, Matilde Cordeiro, Syed Gul A.S. Sani, Sripada M. Udupa, Irshad A. Rather, Reyazul Rouf Mir, Vincent Vadez, George J. Vandemark, Pooran M. Gaur, Douglas R. Cook, Christine Boesch, Eric J.B. von Wettberg, Jana Kholova, R. Varma Penmetsa

**Affiliations:** 1 International Crops Research Institute for the Semi-Arid Tropics (ICRISAT), Patancheru 502 324, India; sakthibiotechbdu@gmail.com (K.S.); V.Vadez@cgiar.org (V.V.); P.Gaur@cgiar.org (P.M.G.); 2 Department of Plant and Soil Science, University of Vermont, and Gund Institute for the Environment, Burlington, VT 05405, USA; Edward.Marques@uvm.edu (E.M.); Erika.Bueno@uvm.edu (E.B.);; 3 School of Food Science and Nutrition, University of Leeds, Leeds, LS2 9JT, UK; fsnak@leeds.ac.uk (N.K.); C.Bosch@leeds.ac.uk (C.B.); 4 Department of Plant Pathology, University of California, Davis, CA 95616, USA; noecarras@ucdavis.edu (N.C.-G.); peterc@usc.edu (P.L.C.); embergmann@ucdavis.edu (E.M.B.); matilde.cordeiro@gmail.com (M.C.); drcook@ucdavis.edu (D.R.C.); 5 International Center for Agricultural Research in the Dry Areas (ICARDA), P.O.Box 6299, Rue Hafiane Cherkaoui, 10112 Rabat, Morocco; S.Udupa@cgiar.org; 6 Division of Genetics & Plant Breeding, Sher-e-Kashmir University of Agricultural Sciences & Technology (SKUAST), Sopore 193 201, India; ratherirshad@gmail.com (I.A.R.); imrouf2006@gmail.com (R.R.M.); 7 Grain Legume Genetics and Physiology Research, USDA-ARS, and, Washington State University, Pullman, WA 99164, USA; George.Vandemark@ars.usda.gov; 8 Department of Plant Sciences, University of California, Davis, CA 95616, USA

**Keywords:** Mendel’s I gene, cosmetic stay-green, biofortification, green cotyledon, carotenoids, pro-vitamin A, chickpea, *Cicer arietinum*

## Abstract

“Stay-green” crop phenotypes have been shown to impact drought tolerance and nutritional content of several crops. We aimed to genetically describe and functionally dissect the particular stay-green phenomenon found in chickpeas with a green cotyledon color of mature dry seed and investigate its potential use for improvement of chickpea environmental adaptations and nutritional value. We examined 40 stay-green accessions and a set of 29 BC2F4-5 stay-green introgression lines using a stay-green donor parent ICC 16340 and two Indian elite cultivars (KAK2, JGK1) as recurrent parents. Genetic studies of segregating populations indicated that the green cotyledon trait is controlled by a single recessive gene that is invariantly associated with the delayed degreening (extended chlorophyll retention). We found that the chickpea ortholog of Mendel’s I locus of garden pea, encoding a SGR protein as very likely to underlie the persistently green cotyledon color phenotype of chickpea. Further sequence characterization of this chickpea ortholog CaStGR1 (CaStGR1, for carietinum stay-green gene 1) revealed the presence of five different molecular variants (alleles), each of which is likely a loss-of-function of the chickpea protein (CaStGR1) involved in chlorophyll catabolism. We tested the wild type and green cotyledon lines for components of adaptations to dry environments and traits linked to agronomic performance in different experimental systems and different levels of water availability. We found that the plant processes linked to disrupted CaStGR1 gene did not functionality affect transpiration efficiency or water usage. Photosynthetic pigments in grains, including provitaminogenic carotenoids important for human nutrition, were 2–3-fold higher in the stay-green type. Agronomic performance did not appear to be correlated with the presence/absence of the stay-green allele. We conclude that allelic variation in chickpea CaStGR1 does not compromise traits linked to environmental adaptation and agronomic performance, and is a promising genetic technology for biofortification of provitaminogenic carotenoids in chickpea.

## 1. Introduction

The chickpea is an important source of nutrition and economic livelihood for developing countries [1]. In developing semiarid tropical (SAT) regions, chickpea is typically grown during the post-rainy season under rain-fed conditions [2]. As a result of this growing practice, fluctuations in crop yields largely reflect in-season water availability and crop adaptation to these conditions. Fluctuations in crop production threaten the nutritional and economic status of the already impoverished smallholder farming communities, which make up 80% of all Asian and African farmers [3]. One way to alleviate chickpea production fluctuations in SAT is through the introduction of cultivars with enhanced climate resilience and nutrient density. The utilization of functional stay-green phenotypes is a possible solution to enhance crops climate resilience due to its ability to conserve water and nutrients in drought conditions [4]. Functional stay-green technology is extensively studied and exploited by many crop improvement programs (mainly in cereals, sorghum: [5,6,7,8,9,10]; maize: [11,12,13,14]; wheat: [15,16,17,18,19]; rice: [20,21,22,23].

The biological basis (i.e., plant constitutive water and nutrient use dynamics) and benefits of the functional stay-green trait for the SAT agrisystems have been well documented [10,24,25,26,27,28]. In contrast, cosmetic-stay green which is linked to naturally occurring loss-of-function allelic variants [29] with dysfunctional chlorophyll degradation pathways, has rarely been studied in these conditions. This type of stay-green results in extended retention of chlorophyll in all plant organs (leaves, stems, grains) and delays age-related senescence as well as senescence caused by environmental factors (e.g., drought). The utility of cosmetic stay-green variants has been, thus far, limited to green color retention in ornamentals, vegetables, and turf-grasses [29]. However, green-seeded variants also occur in many legumes and pulses such as, chickpea, common bean, lima bean, lentil, cowpea, and pea. Seed greenness in pea has resulted into two major market categories, yellow and green pea, demonstrating the vast economic potential of this trait in other legumes and pulses.

The cosmetic stay-green trait might have much more practical implications than just visual appearance caused by extended chlorophyll retention [29,30,31,32,33]. For example, it is well known that chlorophyll biosynthesis and retention is co-regulated with carotenoids which facilitate scavenging of reactive oxygen species generated in the process of photon’s capture by chlorophylls [34,35,36]. Therefore, we may expect that extended chlorophyll maintenance in any plant organ (including seeds) to be associated with extended maintenance of carotenoids (including β-carotene, i.e., provitamin A), which are of relevance to improving the human diet [37,38] as observed in chickpea [30,39]. On the other hand, the retention of chlorophyll and its associated pathways in cosmetic stay-green crops may impose drawbacks on crop agronomic performance, such as slow seedling establishment or arrested N-remobilization [29,40,41,42,43,44,45,46,47].

Therefore, in this study we aim to characterize the genetic, molecular and physiological basis of cosmetic stay-green trait in chickpea. We document allelic variation in the chickpea ortholog of the ‘staygreen’ protein that is invariantly associated with genotypes of the green cotyledon color. We test the functional consequences of ‘stay-green’ on several key plant processes linked to water usage, transpiration efficiency, and other agronomic traits important for chickpea production in drought-prone regions of the semiarid tropical (SAT) agrisystems. Lastly, we examined stay-green’s potential for natural biofortification of chickpea to alleviate the nutritional deficiencies commonly found in these systems.

## 2. Results

### 2.1. Delayed Degreening Phenotypes in Green Cotyledon Chickpea and Underlying Allelic Variation

#### 2.1.1. Delayed Degreening Phenotypes in Green Cotyledon Chickpea

**Figure 1:**
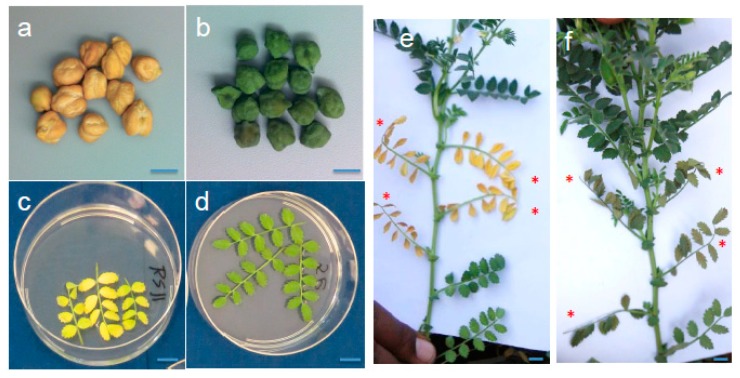
Seed and leaf senescence phenotypes of normal and green chickpea. Dried mature seed of common chickpea with yellow cotyledons (**a**) and of the green cotyledon colored type (**b**). Differential degreening rates in detached leaves floated on water after 5 days in the absence of light from normal chickpea (**c**) and green cotyledon type (**d**), and from leaves wrapped in aluminum foil from yellow (**e**) and green chickpea (**f**). Asterisk in (**e**) and (**f**) mark leaves covered by foil for 5 days. Blue lines in each panel correspond to 1 cm.

In the initial examination of two green-seeded accessions, PI 450,727 and W6 25975, we observed a delay in degreening of mature plant tissues after harvesting, including of leaves and pods. Subsequent senescence assays of fresh growing leaves (Figure 1) corroborated the initial observations of delayed degreening that were made on harvested whole plants.

To determine the extent of co-occurrence of delayed degreening of leaf tissues and the green cotyledon trait, we examined degreening in a broader set of green cotyledon chickpea. Using the detached leaf assay, examined degreening among 30 green-seeded chickpea germplasm available from the public gene banks, alongside four other germplasm lines with yellow cotyledon color (Table S1). In this experiment all 27 green cotyledon accessions (three other accessions did not germinate) exhibited delayed degreening, with detached leaves remaining remained visually green through day 7 of the detached leaf assay (Table S1). By contrast, each of the four yellow cotyledon accessions exhibited an apparently normal degreening phenotype, with progressive yellowing of leaves clearly evident by day 7 after the start of the experiment (Figure 1c,d). Furthermore, in a separate experiment, we examined degreening of leaves of this germplasm accessions using an “on-planta” assay, wherein leaves were wrapped in aluminum foil (to block out light and trigger degreening) and degreening assayed 5–10 days later (Figure 1e,f). Of 29 accessions assayed in this manner, all 26 green cotyledon lines exhibited persistent green leaves, while by contract, the three yellow cotyledon accessions exhibited yellow-colored leaves (Table S1). Together, the data from the two different assays for degreening invariantly correlated green cotyledon seed types with delayed degreening (senescence) of leaf tissues, and which contrasted with a more rapid (normal) senescence of leaves of the yellow cotyledon seed types. Moreover, this association held true in additional genotypes (breeding lines or cultivars) that were analyzed subsequently (Supplementary Table S1).

#### 2.1.2. Identification of Chickpea Ortholog of the Staygreen (SGR) Protein

**Figure 2:**
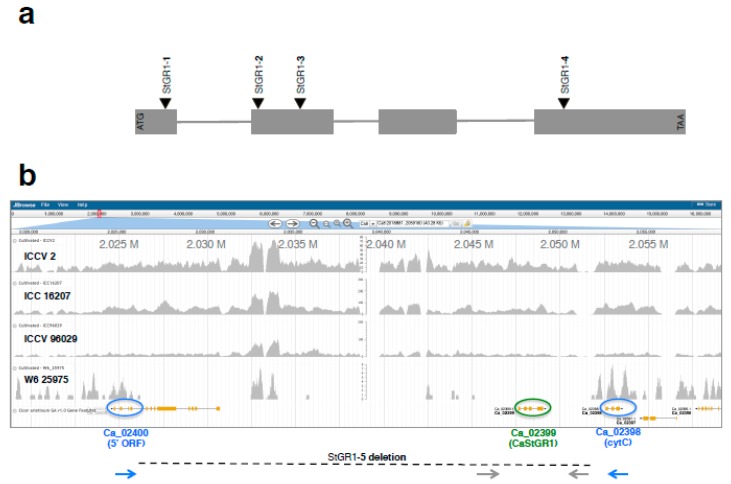
Gene structure and genomic context of type chickpea stay-green gene CaStGR1. (**a**) Schematic of the gene structure of CaStGR1 are shown in (**a**), with the four exons denoted by gray boxes and the three introns as thin lines. Locations of the four small deletion alleles CaStGR1 through CaStGR4 are denoted by triangles above the exons. (**b**) Whole genome Illumina short read skim sequencing read pileups of three normal yellow cotyledon colored chickpea genotypes (ICCV 2, ICC 16,207 and ICCV 96029) are aligned to the chickpea reference of ‘CDC Frontier’, alongside those from genotype W6 25,975 that harbors the large deletion allele CaStGR1-5. Predicted genes Ca-02399 (CaStGR1) and two flanking low copy genes Ca-02398 (cytC) and Ca-02400 (5′ ORF) are marked by ovals. Location of oligonucleotides used in PCR amplification assays from the vicinity of CaStGR1 and falling within the large deletion are marked by gray arrows, and those from the deletion spanning amplification PCR are marked by blue arrows.

The delayed degreening observed to be associated with the green cotyledon colored chickpea was reminiscent of the ‘stay green’ phenotype. This suggested that the ‘staygreen’ gene as a potential candidate gene in chickpea, as this protein has been previously shown to underlie the green-cotyledon trait at Mendel’s I locus in garden pea [48,49]. To identify chickpea sequence homologs of SGR protein, coding regions of SGR protein from pea and Medicago [33] were used in blast searches to identify chickpea transcript assemblies and genomic sequences from public databases. Alignment of messenger RNA sequences against the genomic sequence of chickpea indicated a gene structure comprised of four exons interspersed with three introns (Figure 2a and Supplementary Figure S1). Oligonucleotide primers were designed to encompass the entire coding region of the STG gene and used for PCR amplification from cDNA and genomic DNAs of yellow cotyledon chickpea. Amplified PCR products were Sanger sequenced and aligned against the transcript and genomic sequences of chickpea. The 100% correspondence of the sequence between the amplicons and those of the reference transcriptome and genomic sequences of chickpea confirmed the on-target amplification of the chickpea homolog. We designated this gene as CaStGR1 (for *Cicer arietinum Stay-Green* gene 1).

#### 2.1.3. Association of CaStGR1 Sequence Variants with Green Cotyledon Chickpea Germplasm

Examination of the nucleotide sequence of the green-cotyledon line PI 450,727 indicated a single nucleotide (1-bp) deletion within the first exon of CaStGR1. This frameshifting mutation is predicted to result in missense changes (from amino acid residue 34) coupled with premature termination of translation (at amino acid residue 56) of 266 amino acid residues of a full-length, functional ‘wild type’ CaStGR1 protein.

To determine the prevalence of delayed degreening and of nucleotide variation in CaStGR1 more broadly among chickpea germplasm, we examined the rate of degreening in a set of 53 chickpea lines in total (Supplementary Table S1). This collection was predominantly germplasm from the US gene bank (34 accessions) that was supplemented with breeding lines (15 genotypes) and cultivars with green cotyledon color, with a smaller number of normal, tan/yellow cotyledon lines serving as controls (Supplementary Table S1).

A total of 33 genotypes of which 27 possessed green cotyledons, including genotypes PI 450,727 and W6 25,975 which were analyzed previously, along with six additional genotypes with yellow cotyledons, were assessed phenotypically in a leaf degreening assay. In this analysis, all of the 27 green cotyledon genotypes exhibited delayed degreening, whereas by contrast, all six of the yellow cotyledonary lines senesced rapidly with yellowing of detached leaves by day five of the experiment. Furthermore, the degreening phenotype of the 27 with green cotyledons were indistinguishable from that of the previously characterized genotypes PI 450,727 and W6 25,975 that were included alongside in this analysis. This invariant association between green cotyledon color and delay in degreening of detached leaves suggested that the additional 25 germplasm lines may harbor similar molecular variation previously observed in genotypes PI 450,727 and W6 25975.

PCR amplification with CaStGR1-specific oligos with genomic DNA as the template was conducted in 41 genotypes, of which 37 were green cotyledonary with the remaining four with yellow cotyledons. Amplification was consistently unsuccessful in 10 green cotyledon genotypes despite exhaustive PCR attempts, in a manner similar to that in the presumptive large-deletion in genotype W6 25,975 (Supplementary Table S1). Sanger sequencing of PCR amplicons revealed the presence of the 1-bp deletion previously identified in genotype PI 450,727 in an additional six genotypes (Supplementary Table S1 and Supplementary Figure S1). We designated this variant as CaStGR1-1 allele. Of the remaining 25 genotypes, the four genotypes with yellow cotyledons each had a nucleotide identical to that of ‘wild type’ staygreen gene (that we designated as allele CaStGR1), whereas the remaining 21 genotypes with green cotyledons contained either one of three nucleotide variants in the coding region of the CaStGR1 gene (Supplementary Table S1). Accession ICC 16,340 that was used as the source for breeding of green cotyledon chickpea at ICRISAT-India, along with four breeding lines (also from the ICRISAT-India chickpea breeding program) all shared a novel 8-bp deletion in exon 2 (Supplementary Table S1 and Figure S1) that we designated as allele CaStGR1-2. Ten other genotypes (9 germplasm accessions and the Canadian green-cotyledon cultivar “CDC Verano”) shared another molecular lesion, consisting of a 1-bp deletion (Supplementary Table S1 and Supplementary Figure S1) that we designated as allele CaStGR1-3. Although this variant is also located within exon 2 of CaStGR1, it falls downstream in the coding sequence of the location of the 8-bp deletions observed among material from ICRISAT (allele CaStGR1-2; Supplementary Figure S1). The remaining six green cotyledon genotypes, that included three germplasm accessions and three breeding lines from the USDA-ARS breeding program in Pullman, Washington, USA, each harbored yet another molecular variant, in the form of a 1-bp deletion in exon 4 of CaStGR1 (Supplementary Table S1 and Supplementary Figure S1) which we designated as allele CaStGR1-4). Taken together, the PCR amplification and amplicon sequencing data identified five different molecular lesions in CaStGR1 (Figure 1a and Supplementary Figure S1) that occur exclusively among green cotyledon genotypes (Table 1 and Supplementary Table S1).

#### 2.1.4. Whole Genome Skim Sequencing Delimits the Extent of the Deletion in Allele CaStGR1-5

The absence of amplification in genotypes with the CaStGR1-5 allele with oligonucleotide primers located within the entire coding regions of CaStGR1 was suggestive of a larger sized deletion. To characterize the extend of this deletion we focused on genotype W6 25,975 that typifies this large-deletion allele. In an initial experiment, using the draft whole genome of chickpea genotype CDC-Frontier [50] as a guide, oligos sited in low copy sequences immediately adjacent (within few kbp) to CaStGR1 were designed and used in PCR amplification. Amplification products of the expected size (3-6 kbp in length) were consistently obtained from wild type ICCV 96,029 genotype and PI 450,727 harboring a 1-bp in exon 1 (allele CaStGR1-1). By contrast, no amplification products were obtained from W6 25975, indicating a deletion of larger and yet to be determined size.

To further characterize the extent of this deletion, a whole genome shotgun library was prepared using genomic DNA of the green cotyledon genotype W6 25,975 and sequenced with Illumina HiSeq platform. Sequences obtained were aligned against short read data from normal yellow cotyledon genotypes ICCV2, ICC 16,207 and ICCV 96029, and anchored to the draft whole genome sequence of chickpea genotype CDC-Frontier [50]. Analysis of the resulting pileup of short-read data localized the wild type CaStGR1 gene to between positions 2.047 and 2.049 Mbp on chickpea chromosome 8′s pseudomolecule (Figure 2b). This multi-genotype sequence pileup data suggested a deletion of ~25 kbp in length, from ~2.026 Mbp within an adjacent predicted gene on one side, through CaStGR1 at ~2.047 Mbp, and into another predicted gene at ~2.052 Mbp on the other side of CaStGR1 (Figure 2b). Oligonucleotide primers were designed in the low copy predicted genes at ~2.026 Mbp and ~2.052 Mbp that flank CaStGR1, to encompass the ~25 kbp deduced deletion. PCR amplification with these deletion-spanning oligos yielded amplification products of the expected size (3–6 kbp) in genotype W6 25,975 but not in PI 450,727 (where the amplicon would be >25 kbp in size, beyond the capacity of PCR conditions used). The whole genome skim sequencing data together with the PCR results with the gap-spanning oligos corroborate that the CaStGR1-5 allele represents a large deletion of ~26 kbp in size that encompasses the entirety of the CaStGR1 gene (Figure 2b).

#### 2.1.5. Genetic Cosegregation of Staygreen Sequence Variants with the Green-Cotyledon Trait

In two F2 populations that we examined, the green cotyledon trait segregates as a monogenic recessive trait. In the PI 450,727 x RS11 F2 population, of 47 F2s 35 were of yellow cotyledon color with the remaining 12 with green cotyledon color. In a second F2 population of 88 individuals derived from a cross between yellow cotyledon cultivar ‘Royal’ and the green cotyledon accession PI 359555, 63 F2s had yellow cotyledons and the remaining 25 F2s had green colors. These fit the 3:1 ratio that is expected for a monogenic recessive gene in the F2 generation (with chi-square values of 0.007 and 0.545; and *p*-values 0.933 and 0.460 for the PI 450,727 x RS11 and Royal x PI 359,555 F2 populations respectively).

The single nucleotide deletion identified in the green cotyledon accession PI 450,727 creates a Hpy-188I restriction enzyme recognition site, which allowed for the design of a CAPS (cleaved amplified polymorphic sequence) marker for the CaStGR1-1 variant allele. A F2 population of 47 individuals, derived from a cross between PI 450,727 (with green cotyledons) and accession RS11 (with normal yellow cotyledons), was phenotyped for cotyledon color and genotyped with the Hpy-188I CAPS marker. In this analysis, all 12 F2 individuals with green cotyledons were homozygous for the PI 450,727 allele, while the remaining 35 F2 individuals were either heterozygous or homozygous for the yellow cotyledon allele of RS11, as would be expected for a monogenic recessively inherited trait conditioning green cotyledon color.

We further examined cosegregation between cotyledon color and molecular variation in the CaStGR1 gene in additional F2 populations. A green cotyledonary breeding line with the CaStGR1-4 allele was crossed to the elite cultivars ‘Nash’ and ‘Billybean’ from which F2 populations were generated. Seeds of these F2s were scored for cotyledon color prior to sowing, and subsequently degreening of vegetative leaves assessed by the foil wrap assay. A KASP marker assay for the 1-bp deletion that occurs in this allele was developed and used to genotype these F2 individuals, and to examine the correlation with the seed cotyledon color and degreening phenotypes. In this analysis, all 52 individuals with green cotyledons and delayed degreening of leaves were homozygous for the 1-bp deletion allele. Of an additional 55 individuals with yellow cotyledons and rapid degreening of leaves, 24 individuals were homozygous for the wild type allele, with the remaining 31 individuals heterozygous for the two alleles. These observations are consistent with the expected monogenic recessive nature expected for the CaStGR1-4 allele. The loss-of-function of the protein in the 52 homozygotes for the deletion allele engendering phenotypes on seed color. By contrast, the presence of one or more of the wild type alleles in the other 55 individuals provides a functional protein, and the associated normal yellow cotyledon color and normal rate of degreening.

### 2.2. Characterization of Physiological Functions of Green Cotyledon Chickpea

The genetic and early phenotypic analysis indicated that green cotyledon chickpea is sharing a common suite of characteristics such as delayed degreening in leaf tissue, and which were in contrast to those observed in regular yellow cotyledon chickpeas. To determine the impacts of altered function of the chickpea stay-green gene in these green cotyledon lines, we undertook a set of studies to characterize the impacts on plant physiological functions and indicators of agronomic performance.

#### 2.2.1. Plant Responsiveness to Soil and Atmospheric Drought (Experiment 1 and 2)

**Figure 3:**
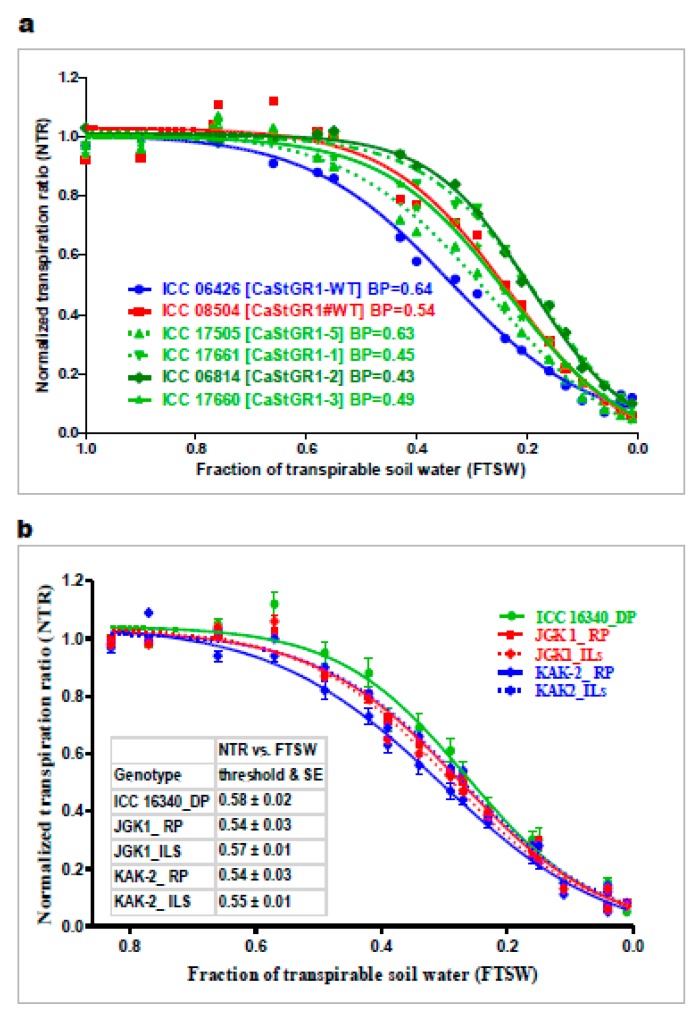
(**a**) Normalized transpiration ratio (NTR) versus fraction of transpirable soil water (FTSW) of chickpea genotypes differed in deletion of CaStGR1gene segments [ICC 08504-CaStGR-1-#Wild type (filled square with solid red line); ICC 06426-CaStGR-1-Wild type (filled round with solid blue line); ICC 17505-CaStGR-1-5 (filled upward triangle with dashed green line); ICC 17661-CaStGR-1-1 (filled down-word triangle with dashed green line); ICC 06814-CaStGR-1-2 (filled diamond with solid green line) and ICC 17660-CaStGR-1-3 (open round with solid green line)] exposed to progressive drying soil under glasshouse conditions. During detached leaf green assay, ICC 08504-CaStGR-1-#Wild type showed yellow colour in all leaflets fully. By contrast, ICC 06426-CaStGR-1-Wild type showed semi-green colour leaflets. Genotypes with CaStGR1-1 (ICC 17661), CaStGR1-2 (ICC 06814), CaStGR1-3 (ICC 17660), and CaStGR1-5 (ICC 17505) showed completely green colour in all the leaflet during detached leaf green assay. Values are transpiration data of five replicated plants for each genotype at each FTSW condition. The FTSW thresholds where transpiration initiated its decline were calculated with a plateau regression procedure from SAS. The regression lines of the relationships between NTR and FTSW were drawn by fitting NTR to FTSW data above and below the respective threshold for transpiration decline in each genotype with GraphPad Prism. The FTSW breakpoint (BP) are displayed in the figures. (**b**) Normalized transpiration ratio (NTR) versus fraction of transpirable soil water (FTSW) of stay green chickpea introgression lines (ILs) with different genetic background [stay green donor parent (DP) ICC 16,340 (square with solid green line); Recurrent parent (RP) JGK1 (square with solid red line); JGK1 background introgression lines JGK1-ILs (square with dashed red line); Recurrent parent (RP) KAK2 (diamond with solid blue line); KAK2 background introgression lines KAK2-ILs (diamond with dashed red line)] exposed to progressive drying soil under glasshouse conditions. Values are transpiration data of five replicated plants for each genotype at each FTSW condition. The FTSW thresholds where transpiration initiated its decline were calculated with a plateau regression procedure from SAS. The regression lines of the relationships between NTR and FTSW were drawn by fitting NTR to FTSW data above and below the respective threshold for transpiration decline in tested genotype with GraphPad Prism. The FTSW breakpoint (BP) and their confidence intervals of regressions are displayed in the figures.

The main purpose of the response to soil and atmospheric drought experiments (experiment 1 and 2) was to characterize the crop capacity to restrict transpiration upon severing soil/atmospheric moisture deficit. The plant responsiveness to soil moisture deficit could be expressed as the soil moisture threshold (i.e., fraction of transpirable soil water; FTSW) when the plant transpiration significantly declines compared to transpiration of WW plants. Across the experiments, we documented a wide range of the genotypic responses to declining soil moisture. FTSW values of 0.43–0.64 were observed among germplasm (Figure 3a), which encompassed the narrower range of FTSW (0.54-0.58) observed in stay-green introgression lines (ILs) that originated from the Indian elite cultivars KAK2 and JKG1 (Figure 3b and Table 2). Within the germplasm lines, genotypes with functional StGR1-WT allele tended to limit their transpiration at a higher level of soil moisture (FTSW threshold higher than 0.5) although we couldn’t statistically differentiate these lines from the other tested StGR1 allelic variants. In the series of experiments with introgression lines (ILs) based on Indian elite cultivars (KAK2 and JKG1), we found that FTSW thresholds of both cultivated recurrent parents (KAK2 and JKG1) was very narrow (0.54 ± 0.03) and significantly lower compared to the FTSW of the stay-green trait donor parent ICC 16,340 (0.58 ± 0.02) whereas there was no significant difference between ILs and the parental lines.

**Figure 4:**
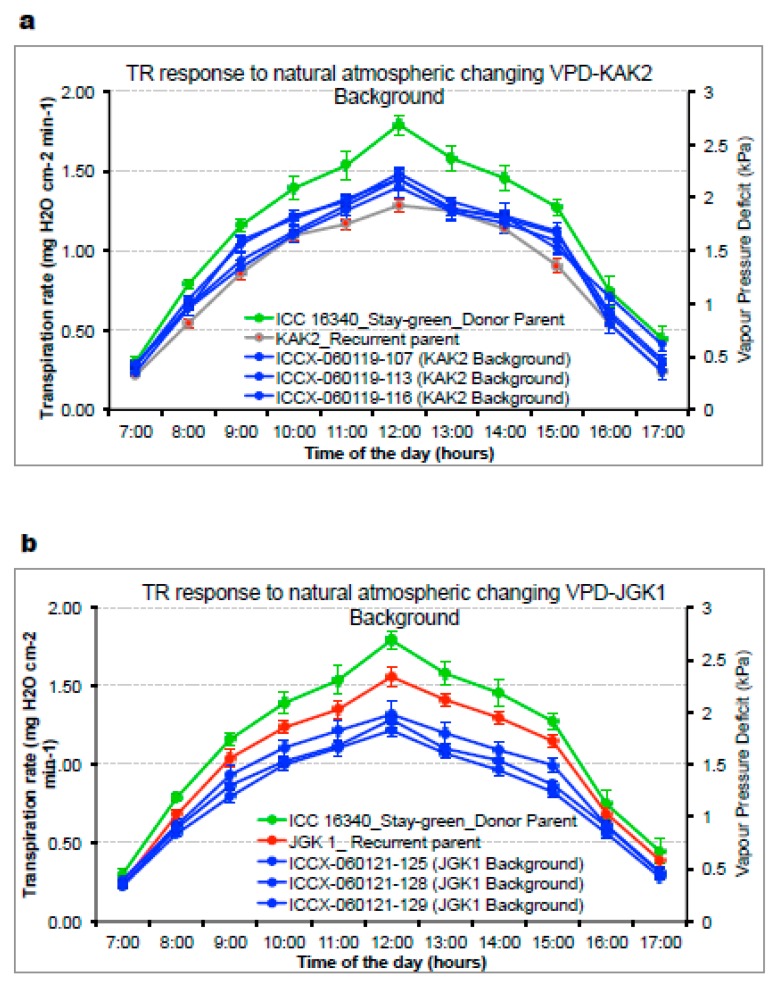
Transpiration rates (TR) of stay green chickpea introgression lines (ILs) with different genetic backgrounds of KAK2 elite cultivar (**a**), and 4JGK1 elite cultivar (**b**). Stay green donor parent (DP) ICC 16,340 (round with solid green line); Recurrent parent (RP) KAK2 (round with solid red line); KAK2 background introgression lines ICCX-060119-107, ICCX-060119-113, ICCX-060119-116 and ICCX-060119-123 (round with solid blue line); Recurrent parent (RP) JGK1 (round with solid red line); JGK1 background introgression lines ICCX-060121-125, ICCX-060121-128 and ICCX-060121-129 (round with solid blue line)] are response to natural changing in the atmospheric vapour pressure deficit (VPD) cycle. TRs were measured on well-watered plants grown in the glasshouse, which were temporarily transferred to outdoor conditions. There, plants were exposed to natural changing atmospheric VPD. TR and VPD data were used to draw a segmental or a single linear regression for all tested genotypes. Each data points represents the means (± SE) of eight replicates per genotype.

**Figure 5:**
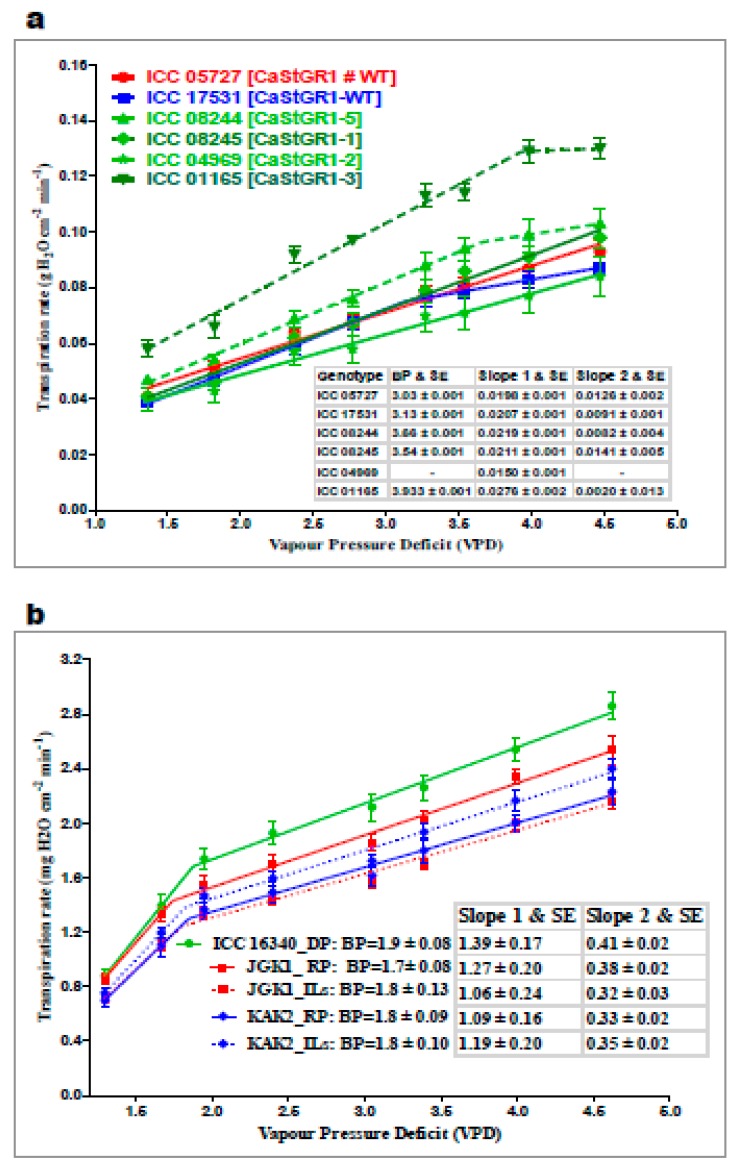
(**a**) Transpiration rates (TR) of six selected chickpea genotypes differed in deletion of CaStGR1gene segments [ICC 05727-CaStGR-1-#Wild type (round with solid red line); ICC 17531-CaStGR-1-Wild type (square with solid pink line); ICC 08244-CaStGR-1-5 (upward triangle with solid green line); ICC 08245-CaStGR-1-1 (diamond with solid blue line), ICC 04969-CaStGR-1-2 (star with solid orange line) and ICC 01165-CaStGR-1-3 (downward triangle with solid pink line)] in response to increasing VPD. During detached leaf green assay, ICC 05727-CaStGR-1-#Wild type showed yellow colour in all leaflets fully. By contrast, ICC 17531-CaStGR-1-Wild type showed semi-green colour leaflets. Genotypes with CaStGR1-1 (ICC 08245), CaStGR1-2 (ICC 04969), CaStGR1-3 (ICC 01165), and CaStGR11-5 (ICC 08244) showed completely green colour in all the leaflet during detached leaf green assay. TRs were measured on well-watered plants grown in the glasshouse, which were temporarily transferred to a growth chamber with control over temperature and relative humidity. There, plants were exposed to increasing VPD, set by modifying temperature and humidity. TR data are the mean of five replicate plants, computed hourly at each of the eight VPD levels. Data were used to draw a segmental or a single linear regression for all tested genotypes. Each data points represents the means (± SE) of five replicates per genotype. The slopes and breakpoint (BP) of regressions are displayed in the figures. (**b**) Transpiration rates (TR) of stay green chickpea introgression lines (ILs) with different genetic background [stay green donor parent (DP) ICC 16,340 (square with solid green line); Recurrent parent (RP) JGK1 (square with solid red line); JGK1 background introgression lines JGK1-ILs (square with dashed red line); Recurrent parent (RP) KAK2 (diamond with solid blue line); KAK2 background introgression lines KAK2-ILs (diamond with dashed red line)] are response to increasing VPD. TRs were measured on well-watered plants grown in the glasshouse, which were temporarily transferred to a growth chamber with control over temperature and relative humidity. There, plants were exposed to increasing VPD, set by modifying temperature and humidity. Data were used to draw a segmental or a single linear regression for all tested genotypes. Each data points represents the means (± SE) of eight replicates per genotype. The slopes and breakpoint (BP) of regressions are displayed in the figures.

Further, we tested the plant’s capacity to regulate transpiration rate (TR [g of water transpired per cm-1 of canopy per h]) in conditions of a drying atmosphere (i.e., increasing vapor pressure deficit; VPD). Here, we documented wide range of variability in the tested material and across the range of conditions (outdoors typically ~0.5–3.0 kPa [Figure 4a,b] and in growth chambers 1.2 to 4.6 kPa [Figure 5a,b]). TR responses to VPD under natural atmospheric (outdoor) conditions and under controlled VPD (growth chamber) conditions showed a similar trend (Figure 4a,b and Figure 5a,b; Table 3). In germplasm, we found no consistent trend in material with (“wild type”) stay-green allele or without (i.e., Loss-of-Function alleles CaStGR1-1 to CaStGR1-5) in the TR responsiveness to VPD (Figure 5a). Some StGR1 loss-of-function germplasm allelic variants were having TR higher while others lower than values observed for germplasm with a functional (wild type) stay-green gene. In experiment 2b and 2c’s series encompassing the stay-green ILs, we found the stay-green donor ICC 16,340 had a higher TR and rapid TR increase upon increasing VPD compared to the recurrent elite parents and their stay-green derivatives in both outdoor and controlled (growth chamber) conditions (Figure 4a,b, Figure 5b; and Table 3). Interestingly, whereas ILs with the KAK2 background had TR and VPD values intermediate to those of the stay-green donor line ICC 16,340 and the elite cultivar KAK2 (Figure 4a), all the stay-green derivatives of JGK1 had even significantly lower TR across the VPD regimes compared to JGK1 elite parent (Figure 4b). Furthermore, in well watered (WW) conditions, there were no significant genotypic differences in the specific leaf weight (SLW) in germplasm, the JKG1-derived ILs had lower SLW compared to both of the parents (Supplementary Figure S2a,b).

#### 2.2.2. Variation in Plant Growth and Water-Use Related Traits in Lysimetric Facility (Experiment 3a, b)

In the lysimeter experiment under well-watered (WW) conditions with germplasm (Experiment 3a) and introgression lines (Experiment 3b), significant genotypic differences in the total amount of water required to reach maturity were observed (data no shown). However this was mostly conditioned by the different phenological development between germplasm and the ILs. The relationship between total water use and days to flowering was strongly correlated in germplasm (R^2^ = 0.63*; Supplementary Figure S3a) but only very weakly in the introgression lines (R^2^ = 0.10ns; Supplementary Figure S3b). Under water stress (WS) differences in total amount of water extracted from lysimeters was independent of crop phenology but these did not coincide with the presence of particular CaStGR1 allele in any of the material used. 

**Figure 6:**
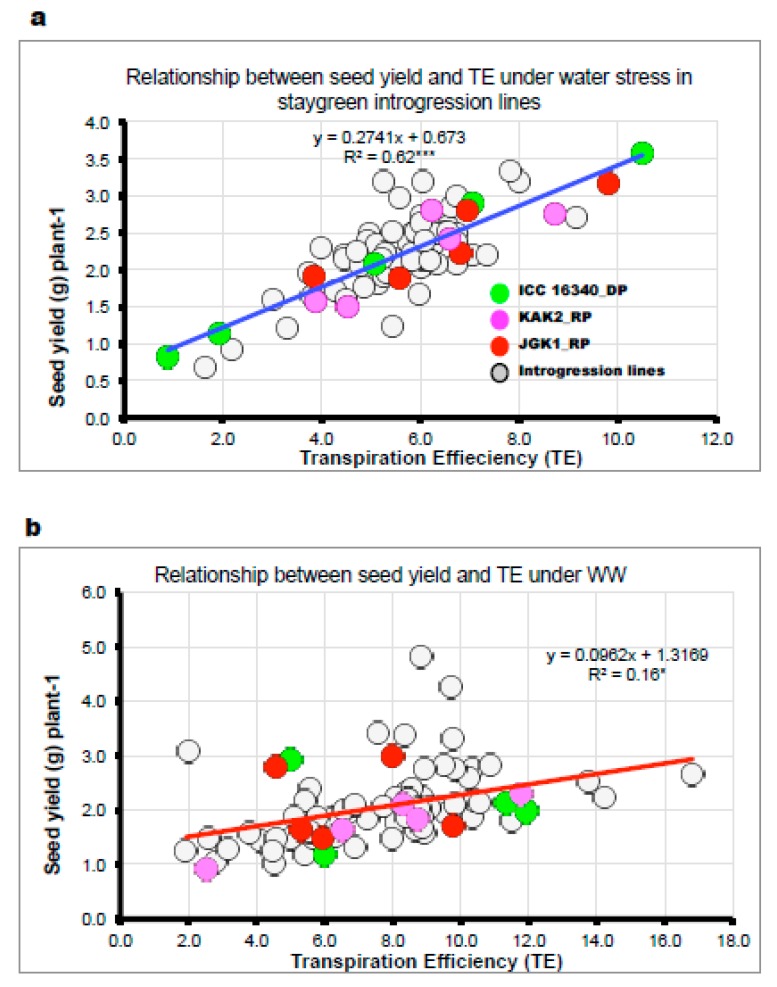
Relationships between seed yield and transpiration efficiency (TE) under (**a**) water stressed (WS) and (**b**) well watered conditions (WW) in stay green chickpea genotypes grown in the PVC cylinders (Lysimetric facility). The data used for these regression analyses are replicated data, obtained under WS and WW conditions. For each genotype, five replicates data points were used to draw the linear regressions. The stay green donor parent (ICC 16340) data are represented in green colour, recurrent parent (JGK1) data are represented in red colour, recurrent parent (KAK2) data are represented in pink colour and introgression lines (ILs) are represented in grey colour. The slopes and R^2^ of regressions are displayed in the figures. R^2^ values with * and *** (astric) symbols are significantly different at *p* < 0.05 and *p* < 0.001.

Under WW and WS, although differences were observed in total biomass accumulation and seed setting, these did not appear to be associated with the stay-green trait. However, the relative decline in total biomass accumulation due to water stress was very similar between all allelic variants with reduction in WS when compared to WW, of ~50% in germplasm and ~30% in IL material. In experiment 3b under WW treatments, the seed yield was largely explained by duration of phenological stages (Supplementary Figure S4). Interestingly in the same experiments under WS, the seed yield did not relate to crops phenology (Supplementary Figure S4) but related positively to seed number (R^2^ = 0.66* in ILs; experiment 3a, R^2^ = 0.73 in germplasm materials). In addition, TE [g biomass per kg of water transpired] and seed yield were strongly associated under WS conditions [R^2^ = 0.62 *** in ILs (Figure 6a) and R^2^ = 0.37 in germplasm ], while there was a weak relationship between TE and seed yield under WW conditions (Figure 6b). Also, in experiment 3b under WS, there were several stay-green isolines in each elite genetic background, which had seed yield comparable or higher than the respective elite recurrents and stay-green donor (Supplementary Figure S5).

#### 2.2.3. Evaluation of Canopy Growth Related Traits (Experiment 4)

**Figure 7:**
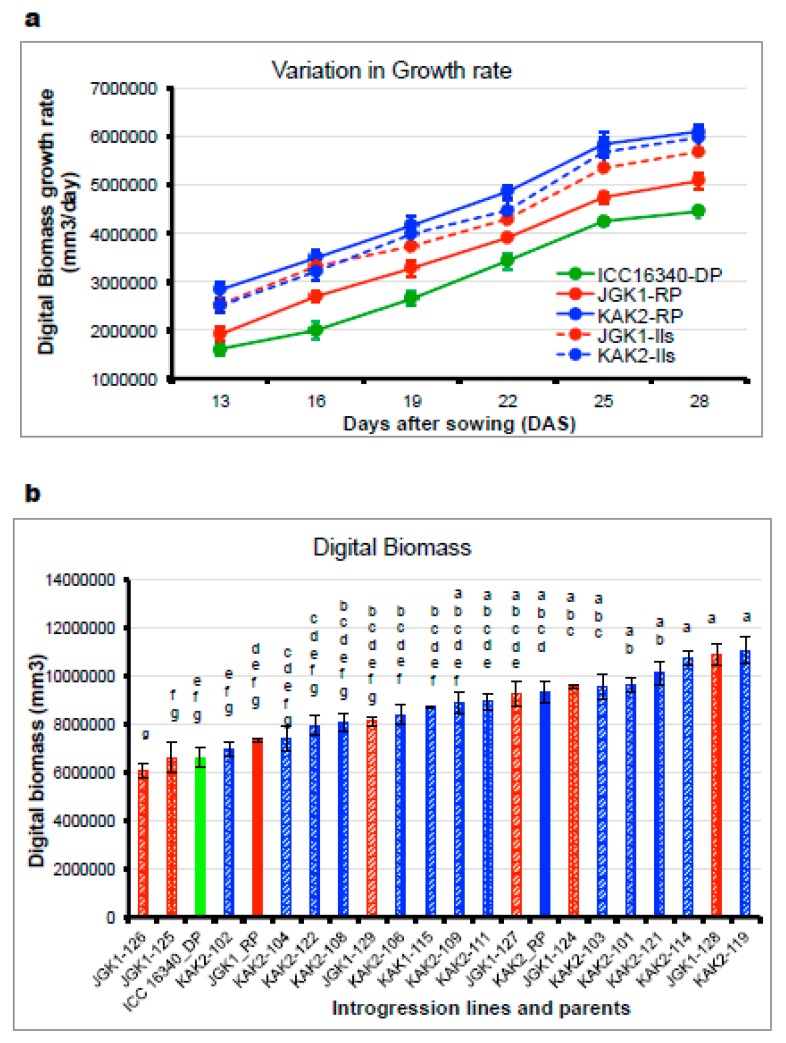
(**a**) Growth rate variation in digital biomass of stay green chickpea introgression lines (ILs) with different genetic background [stay green donor parent (DP) ICC 16,340 (round with solid green line); Recurrent parent (RP) JGK1 (round with solid red line); JGK1 background introgression lines JGK1-ILs (round with dashed red line); Recurrent parent (RP) KAK2 (round with solid blue line); KAK2 background introgression lines KAK2-ILs (round with dashed blue line)] are measured by LeasyScan phenotyping platform. Each data point represents the means (± SE) of four replicates per genotype. Data were used to draw a line graph for all tested genotypes. (**b**) Variation in digital biomass of stay green chickpea introgression lines (ILs) with different genetic background [stay green donor parent (DP) ICC 16,340 (bar filled with solid green colour); Recurrent parent (RP) JGK1 (bar filled with solid red colour); JGK1 background introgression lines JGK1-ILs (bar crossed lines with red colour); Recurrent parent (RP) KAK2 (bar filled with solid blue colour); KAK2 background introgression lines KAK2-ILs (bar crossed lines with blue colour)] are measured by LeasyScan phenotyping platform. Each data points represents the means (± SE) of four replicates per genotype. Data were used to draw a bar graph for all tested genotypes. Bars with different letters are significantly different (*p* < 0.05).

The canopy growth parameters were examined only among stay-green ILs alongside the donor germplasm line ICC 16,340 and the recurrent elite cultivars JGK1 and KAK2. We found the donor parent ICC 16,340 had lower canopy growth rates than elite recurrent parents (JGK1, KAK2) with some of the ILs attaining higher growth rates compared to stay-green donor parent and recurrent parents (Figure 7a) and this reflected in the differences in canopy size and digital biomass estimates averaged across the time of observations (Figure 7b). The parental line JGK1 grew more slowly compared to the elite recurrent line KAK2 (Figure 7a). The stay-green derivative ILs in the KAK2 elite cultivar background had growth rates similar to those of the recurrent elite parent KAK2 (Figure 7a). Growth rates in stay-green ILs originated from the elite cultivar, JGK1 exceeded those observed in both parents, and at levels similar to those of in KAK2 stay-green ILs. This indicated that stay-green IL material had recovered its vigor (Figure 7a).

#### 2.2.4. Evaluation in the Field Conditions (Experiment 5)

**Figure 8:**
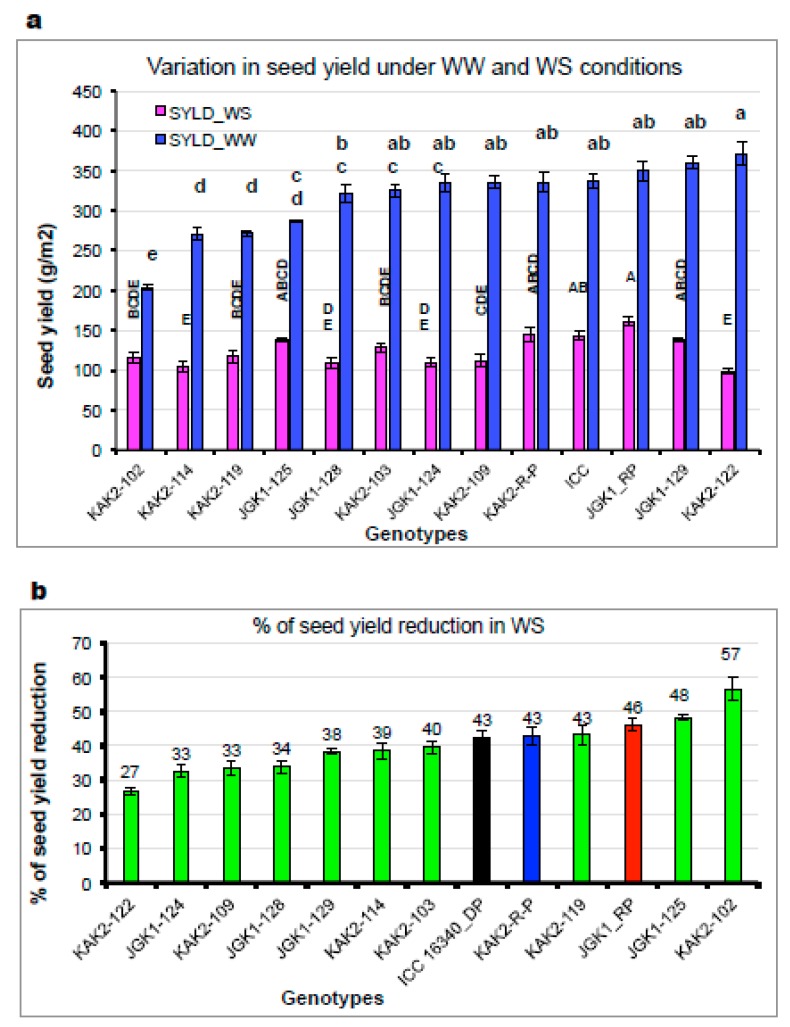
(**a**) Variation in seed yield under well water (bar filled with blue colour) and water stress (bar filled with pink colour) conditions. The data used for these bar graphs are mean data, obtained under well-watered (WW) and water stress (WS) conditions. For each genotype, three replicates data points were used to draw the bar graph. Bars with different capital letters (well-watered—WW) and small letters (water stressed—WS) alphabets are significantly different (*p* < 0.05) and same letters represents non-significant. (**b**) Percentage of seed yield reduction under water stress (WS) conditions. The data used for these bar graph are mean data, obtained from well watered seed yield data were normalised against water-stressed seed yield data and then seed yield reduction values are presented in percentage. The data of stay green donor parent ICC 16,340 (bar filled with black colour); recurrent parent-JGK1 (bar filled with red colour); recurrent parent-KAK2 (bar filled with blue colour); stay-green introgression lines from both JGK1 and KAK2 genetic background–ILS (bar filled with green colour).

The IL plant material that was relatively more homogeneous for the main phenology-related characters was tested in the field alongside their recurrent parents (experiment 5; flowering 37–53 DAS, days to maturity 97-101). Some of the tested ILs attained similar or even higher grain yield under irrigated conditions (Figure 8a), which was partially positively driven by phenology differences [Relationship between accumulated biomass or seed yield and days to flowering; R^2^ = 0.51* (Supplementary Figure S6a) and negatively related to harvest index [Relationship between accumulated biomass and harvest index (HI); R^2^ = 0.30* (Supplementary Figure S6b)]. Water stress (WS) conditions reduced the grain yield cca 40–70%. Under WS conditions, the yield of stay-green ILs in relation with the days to flowering was much looser (Supplementary Figure S6c). We also observed the lack of correlation between the production traits (biomass and yield) and phenology parameters [Regression between accumulated biomass and days to flowering; R^2^ = 0.0001 & regression between seed yield and days to flowering; R^2^ = 0.08] while the relation between HI was maintained [Relationship between seed yield and harvest index (HI); R^2^ = 0.21 (Supplementary Figure S6d)]. Interestingly, we found that the extent of yield reduction due to WS was similar between the parental lines and some of the stay-green introgression line progenies (Figure 8b), and was further positively related to plant capacity to grow in WW but negatively in WS (i.e., higher production potential, higher yield reduction due to WS while the “smaller” plants had suffered less yield reduction under WS).

#### 2.2.5. Leaf Pigments Content Under WW and WS Conditions (Experiments 1c, 3a,b,c)

Pigments in the Leaf Tissues and Grains.

Across all material tested, we found that plants grown outdoors (in lysimeters, experiment 3c) maintained much higher levels of photosynthetic pigments, especially carotenoids in leaves tissues, compared to plants cultivated in the glass-house (in lysimeters, experiment 3b) environments.

**Figure 9:**
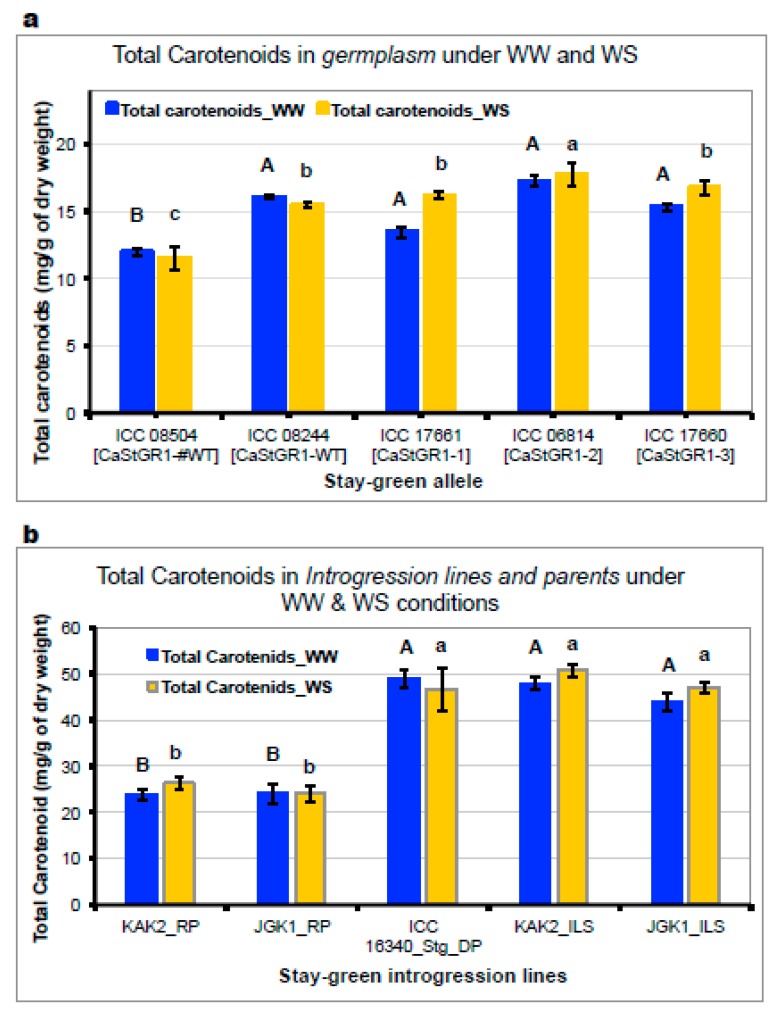
Variation in (**a**) seed total carotenoids content in germplasm [ICC 08,504 (CaStGR1-#WT), ICC 08,244 (CaStGR1-WT), ICC 17,661 (CaStGR1-1), ICC 06,814 (CaStGR1-2) and ICC 17,660 (CaStGR1-3)] and (**b**) stay green chickpea introgression lines (ILs) with different genetic background under well-watered (WW) and water-stressed (WS) conditions. During detached leaf green assay, ICC 08504-CaStGR-1-#Wild type showed yellow colour fully in all leaflets. By contrast, ICC 08244-CaStGR-1-Wild type showed semi-green colour leaflets. Genotypes with CaStGR1-1 (ICC 17661), CaStGR1-2 (ICC 06814) and CaStGR1-3 (ICC 17660) showed completely green colour in all the leaflet during detached leaf green assay In both graph **(a)** and **(b),** closed bars represents WW and open bars are represents WS. Each data points represents the means (± SE) of five replicates per genotype. Data were used to draw a bar graph for all tested genotypes. Bars with different capital letters (well water-WW) and small letters (water stressed-WS) alphabets are significantly different (*p* < 0.05) and same letters represents non-significant.

We found no differences between the levels of leaf pigments (i.e., chlorophyll a, chlorophyll b, total carotenoids) and their ratio (chlorophyll a/chlorophyll b ratio) in the materials carrying the CaStGR1-wt functioning allele and CaStGR1-1 to 5 malfunctioning allele (ILs and some germplasm) under WW. The methodology of stress imposition and the tissue sampling (the last fully developed leaf on the main stem) couldn’t discriminate the stay-green material from wild-type under the WS conditions either. However, we found a higher chlorophyll_a and cholorophyll_b content in mature seeds of material carrying stay-green alleles compared to CaStGR1-wt in both germplasm (Supplementary Figure S7a,b) and stay-green ILs (Supplementary Figure S8a,b). Similarly, the grain total carotenoids content was ~10–30% higher in the stay-green loss-of-function variants (alleles CaStGR1-1 to 5) compared to wild type (CaStGR1-WT; Figure 9a) in germplasm and ILs. Furthermore, grain total caratenoid levels were not significantly affected by the conditions of cultivation (WW and WS) in the introgression lines (Figure 9b).

**Figure 10:**
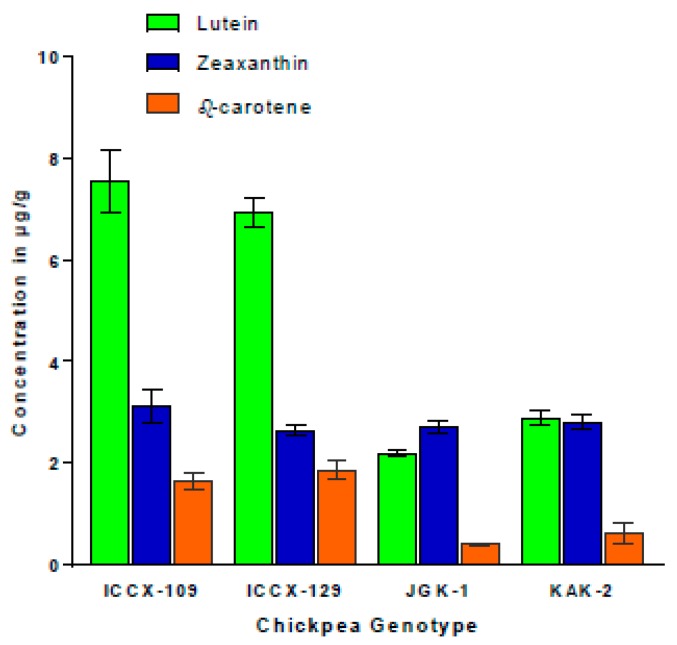
Variation in different carotenoids content (Lutein, Zeaxanthin and beta carotene) in seeds of stay green chickpea introgression lines (ILs) with different genetic background [ICCX-109 (KAK2 genetic background and ICCX-129 (JGK1 genetic background) and their recurrent parents (JGK1 and KAK2). The lutein pigment data are represents in light grey colour bars; Zeaxanthin pigment data are represents in black colour bars and beta carotene pigment data are represents in dark grey colour bars. Each data points represents the means (± SE) of three replicates per genotype.

The detailed fractionation of carotenoids contents in ILs seeds revealed that there were ~3-fold higher levels of lutein and beta-carotene (provitamin A) in the seeds of green cotyledon introgression lines (ILs) compared to both of the yellow cotyledon colored elite cultivars (KAK2 and JGK1; Figure 10). By contrast, zeaxanthin content did not significantly vary between ILs with green cotyledons and the elite cultivars with yellow cotyledons (KAK2 and JGK1; Figure 10) used as recurrent parents in introgression line development.

## 3. Discussion

The two goals of the present study were to (1) understand the molecular and functional mechanisms underlying the delayed senescence in chickpea with the “cosmetic stay-green” trait [29,31] and, (2) to characterize the effects of the “cosmetic stay-green trait” on plant performance in semiarid agricultural systems. Since the majority of chickpea production occurs under water-limited rainfed conditions, (i.e., terminal drought), understanding responses to water limitations is critical to evaluating the potential of stay-green chickpea. Lastly, we also investigated the nutrient composition of stay-green chickpea, as a genetic biofortification technology to alleviate nutritional deficiencies for carotenoids in consumers.

### 3.1. Identification of ‘Cosmetic Stay-Green’ Allele in Chickpea

Recent developments in genome sequencing have provided deep sequence resources for several legumes, in terms of whole genome sequences and transcriptomes. These sequence data provide a valuable resource for both the comparative and evolutionary studies of genome structure and genes. Subsequent analysis of amplified chickpea sequences and their localization to the chickpea draft genome supported the identification of the cognate chickpea stay-green gene that exhibited a high degree of sequence similarity with the other legume stay-green orthologs, and localized to a syntenic position on chromosome 8 in the chickpea draft genome [50]. This genomic region corresponds to the large-effect QTL for carotenoid concentrations described among three F2 populations of chickpea [39], which contains the staygreen gene ortholog (LOC101509366; [39]). Our methods highlight the utility of draft or reference genomes for the more detailed study of individual genes from their initial identification to deduction of orthology from the evolutionary history.

In addition, we also conducted a whole genome skim sequencing, to delimit the extent of the deletion in allele StGR1-5. Initial and exhaustive PCR amplifications indicated this allele as probably encompassing the entire coding region of the chickpea ortholog, but whose boundaries were unknown. The use of whole genome skim sequencing of the genome for this allele allowed us to flank the large (several 10s of kbp; Figure 2b) deletion in a single experiment. This contrasts with earlier approaches such as primer amplicon ‘walking’ that given the large size of the deletion would not have yielded results or required the use of a large collection of oligos at varying distance surrounding the StGR1 gene.

The monogenic recessive nature of the green cotyledon trait is supported by observation of only yellow cotyledon phenotypes in the F1 individuals from crosses between yellow and green cotyledon chickpeas, and in cosegregation data in segregating progenies (described in results). Furthermore, the occurrence of green cotyledon phenotype in F1s obtained from crosses among alleles, and invariantly green cotyledon in their F2s supports our inference that the five molecular variants we identified and describe in this study comprise an allelic series in StGR1 gene.

The recessive behavior of the green cotyledon alleles of chickpea is consistent with a loss-of-function of the chickpea StGR1 gene in these phenotypic variants. This inference is corroborated by the likely impact of the deletions on the deduced amino acid sequence of the translated protein. The single nucleotide deletions in alleles StGR1-1 to allele StGR1-4 all occur within the coding regions, and consequently these deletions would result in a frame shift of the open reading frame (and premature truncation of the translated protein).

The identification of five different loss-of-function alleles in CaStGR1, and the absence of nesting (where more than one deletion allele occurs within a single genotype), implies that the green cotyledon trait arose independently at least four times in chickpea, and as naturally occurring variation among chickpea germplasm. The fifth gene-encompassing deletion allele StGR1-5 could represent a fifth independent origin of green cotyledon trait in chickpea. However, based on our data we cannot preclude the possibility that this allele may have arisen secondarily within the background of one of the other small 1-bp deletion alleles (StGR1-1 to StGR1-4). Additional analyses of the green cotyledon germplasm along with related germplasm might help to clarify this current ambiguity.

It is intriguing that green cotyledon breeding lines from the three different chickpea breeding programs (ICRISAT in India, USDA-ARS in USA, and the University of Saskatchewan in Canada) represent three different and distinct loss-of-function alleles of the stay-green gene as a source of the green cotyledon trait. This could be a reflection of the limited knowledge or availability of the sources of green cotyledon germplasm in these breeding programs. Alternatively, the use of the different alleles in each breeding program might reflect preferential use of distinct germplasm on the basis of other traits (e.g., for local adaptation, market type, disease reactions) present in the various germplasm sources. Indeed, our observation of varying phenology among green cotyledon germplasm could represent such additional phenotypic variation, along with seed size and color that also vary. In such a scenario, the distinct alleles for StGR1 gene are merely inadvertently co-selected for a desired common trait of green cotyledons from germplasm with additional characteristics.

Despite the recurrent selection at an orthologous StGR gene in multiple crop legumes for green cotyledon color, it is possible that additional genes exist that replicate this phenotype, or might modulate it. For example, in the more exhaustively studied Rice and *Arabidopsis* systems (e.g., [29,51,52,53]), genes other than the stay-green protein have also been implicated in the cotyledon color or persistence of chlorophyll machinery which would affect stay-green phenotypes. Furthermore, some aspects of the green cotyledon trait, and its manifestation at the level of whole seeds is also likely to depend on pigmentation in the overlying seed coat tissues. For example, in cowpea, distinct genes controlling green color in cotyledon and green color in seed coats have been described [42,54].

Our identification of the molecular nature of variation among green cotyledon chickpea should facilitate the use of molecular marker assisted selection (MAS) or backcrossing (MABC) for introgression of this trait in chickpea breeding. For example, in the current study we developed and tested a KASP marker for the StGR1-4 allele found in USDA-ARS breeding lines (Supplementary Tables S1 and S2). This assay is effective at monitoring the allele states (wt or 1-bp deletion) within exon 4 of the chickpea gene, and is being used for marker-assisted backcrossing in our program. Design and testing of similar KASP assays for the remaining single nucleotide deletions (alleles StGR1 -1, -2, -3) is being planned to facilitate similar use of MAS with these distinct allelic variants.

### 3.2. Green Cotyledon Trait as a Vavilovian Homologous Series of Variation

Green cotyledon market classes or types occur in several crop legumes, including garden pea [52], Medicago [33], chickpea [30,55], common bean [52], lima bean [52], and cowpea [54]. This recurrence suggests that the green cotyledon color trait arose from the repeated and independent selection from the white or yellow cotyledon forms that typify these crops and their wild relatives. The prevalence of repeated human selection for a common phenotype in multiple crops was suggested by the pioneering crop evolutionary botanist Nikolai Vavilov [56].

### 3.3. Stay-Green Alleles do not Affect the Plant Responsiveness to Soil and Atmospheric Drought

#### 3.3.1. Plant Responsiveness to Soil and Atmospheric Drought

Any novel crop technology intended for practical utilization in complex agrisystems has to be appropriately tested to enhance the probability to be implemented and accepted. In many of the semiarid rain-fed agrisystems, one of the main limiting factors to crop productivity is soil moisture deficit [2,8,57,58,59,60]. To understand plant responses to decreased soil moisture, we have generated substantial evidence on plant functions that contribute to crop adaptations in these environments [61,62,63,64,65]. In the present study we evaluated whether stay-green phenotype in chickpea underlined by CaStGR1 gene might be functionally involved in any important environmental adaptations (i.e., responsiveness to soil and atmospheric drought). We found that in all tested material carrying the stay-green CaStGR1 gene (germplasm or cultivated crop types) we did not observe any association between allelic variation and plant responsiveness to soil/atmospheric drought which would have impacted crop production in dry environments. In the cultivated plant types, we found that CaStGR1-2 stay-green ILs inherited the level of environmental adaptations from the cultivated parent rather than from the donor of this stay-green allele (ICC 16340). In some particular cases, the level of adaptive features was even more pronounced than in the cultivated recurrent parent (JGK-1 and derived ILs; Figure 4b). We speculate that this “transgressive segregation” could have been, at least partially, driven by the higher capacity to grow and expand canopy of ILs originated from this cross (Figure 7b; see [65]).

#### 3.3.2. Plant Water-Use Related Traits and Agronomic Performance

Crop functions linked to quantity and efficiency of water utilization (e.g., see above) determines its agronomic performance, especially in environments limited by the water availability [10,66,67]. As discussed above we showed that CaStGR1 allelic variation does not appear to affect the relatively simple plant functions which were previously documented to influence crop adaptations to dry environments [2,59,68]. However, since crop yield is a very complex trait, we have also tested the CaStGR1 allelic variants in the systems relevant for evaluation of crop agronomic characteristics (i.e., lysimteric system and field).

We found there were significant differences in grain and biomass yield in germplasm when tested under different irrigation regimes but none of the differences seemed to coincide with the presence of disrupted CaStGR1 allele (CaStGR1-1 to CaStGR1-5). These differences in germplasm production characteristics were mostly explained by the differences in phenological development. In the stay-green CaStGR-1-2 ILs derived on cultivated background, we found significant genotypic differences in the main production parameters with the recurrent parents attaining generally higher production (example on Figure 8a). Nevertheless, in each of these experiments there were at least few ILs in the genetic background of each of the two elite cultivars whose production was comparable to the elite recurrent parents under WW and WS treatment (which ILs were consistent). Interestingly, under WS treatment, yield of some ILs was similar to that of their respective recurrent parents despite the phenological development of these ILs was generally several days longer (~14 days). Further, we found that the relation between seed yield and flowering time was much looser than that of the germplasm (as in [69,70])-especially under WS where this correlation was hardly significant (e.g., Supplementary Figure S6). However, we found that the majority of variation in grain yield and yield components within this material was explained by TE, especially under WS (Figure 6a,b). We can speculate that higher TE in some of the tested ILs could have been the consequence of lower TR and increased transpiration responsiveness of some ILs to VPD (see above and Figure 4b). We can further speculate that the enhanced TE of some tested ILs could be a consequence of yet to be determined mechanisms induced by portions or interactions of genome remaining from the donor genotype since the recurrent background of IL material was not completely recovered at BC4-5:F2 (i.e., ~94–97% of recurrent background recovered).

Collectively these data indicate that across the range of tested conditions there is no significant trade-off between elevated carotenoid content and agronomic productivity. Yields were similar between lines with “wild type” CaStGR1 (with yellow cotyledons) and genotypes with loss-of-function alleles in the CaStGR1 gene (with green cotyledon and delayed degreening phenotypes).

#### 3.3.3. Stay-Green Alleles Extend Retention of Chlorophyll and Provitaminogenic Carotenoids in Grains and Leaves

Several stay-green plant phenotypes have been described in different crops [52]. The common denominator of “stay-green” phenotype can be described as a plant’s capacity to remain green (i.e., maintain chlorophylls) in particular circumstances (reviewed [29,71,72]). In general, we can consider two basic stay-green types; “cosmetic” and “functional”. Cosmetic stay-green is underlined by any mechanism that avoids chlorophylls to degrade—therefore the plant tissues appear green even if desiccated. Functional stay-green is a consequence of plants ability to manage resources during the crop cycle (e.g., water and nitrogen; [8,25,27,28,73,74]).

We present evidence that the green-seeded chickpea material is of a “cosmetic” type and depended on the presence of disrupted CaStGR1 gene, an ortholog of Mendel’s I locus of garden pea (see above), that affects the function of chlorophyll degrading enzyme [48] and resulted in retention of chlorophylls in dried plant tissues (grain and leaf). We were further interested in addressing whether the composition of chlorophylls *a* and *b* and the functionally related pigments (carotenoids) differed among plant tissues (grain and leaves) during a range of circumstances (irrigated and water stress).

Consistently, we found that the levels and the composition of pigments did not significantly differ between genotypes carrying disrupted CaStGR1 gene (allele 1–5) and wild-type under irrigated and even under water stress conditions (probably because for this estimation only the leaves from the top of the plants which still remained green even in wild-type were sampled). Nevertheless, we found that all stay-green genotypes, in general, maintained higher level of pigments in matured grains compared to wild-type in irrigated conditions (similarly in [30]). The pigments in the grain were not significantly affected by the conditions of cultivation (WW and WS) across the range of material tested and the grains produced by plants exhibiting stay-green phenotype had all 10–100% higher chlorophyll and total carotenoids contents compared to the respective wild-type checks (similarly in [30,75]). Further dissection indicated the stay-green ILs contained two to three fold higher levels of specific A-provitaminogenic carotenoids (beta-carotene) resembling or exceeding the levels achieved by “golden-rice” technology [39,76].

Additional studies are required to determine the extent to which these elevated levels of carotenoids translate into enhanced bioavailability of vitamin A for humans, factors influencing consumer acceptance of green cotyledon colored chickpeas as dry grains, and if green cotyledon chickpea may be associated with conditionally-reduced seed germination or seedling establishment as has been observed in some other crop legumes.

## 4. Materials and Methods

### 4.1. Plant Material: Chickpea Germplasm and Breeding Lines

Chickpea genotypes with the common yellow cotyledon color and those with the infrequently occurring green cotyledon color were obtained from gene banks (USDA GRIN in Pullman, Washington, and ICRISAT India) and from chickpea improvement programs (detailed in Supplementary Table S1). In the process of plant grow outs for seed multiplication, the gene bank accessions were visually screened for occurrence and confirmation of green cotyledon color in mature dry seeds. Furthermore, during such grow outs we examined degreening of leaves of this germplasm accessions using a detached leaf assay, wherein leaves were wrapped in aluminum foil (to block out light and trigger degreening) and the pigment loss/retention capacity (“degreening”) assayed 5–10 days later. The same plants were tested for sequence polymorphism in the CaStGR1 gene (see below). In initial germplasm screen, eight lines with green cotyledon color representing four different allelic variants in the chickpea stay green candidate gene and two yellow cotyledon genotypes carrying the wild-type alleles were used in physiological studies (Supplementary Table S1). In these initial studies, as expected [30] elevated levels of total carotenoids among green cotyledon color lines relative to concurrently grown normal yellow chickpea lines was observed.

For the subsequent and more detailed analyses, breeding lines with contrasting yellow and green cotyledon color were used (Supplementary Table S1). These lines were derived from introgression of the green cotyledon trait from the germplasm accession ICC 16,340 into two Indian elite chickpea cultivars, JGK1 and KAK2 with yellow cotyledon colors. 25 BC4-5:F2 generation introgression lines and their parents were screened for phenology and agronomic traits. Based on homogeneous phenology (flowering time, duration of flowering) and agronomical traits (harvest index), genotypes were selected for further studies (Supplementary Table S3) details of genotypes used in different experiments).

### 4.2. Molecular Characterization of Candidate Gene and Genome:

The genotypes tested for variation in CaStGR1 allele are shown in S1 table. In these, the genomic DNA was extracted from the young leaflets using QIAGEN DNeasy Plant Kit following the manufacturer’s recommended procedures, or from seed-derived cotyledon tissue (for the cultivar ‘CDC Verano’) using a phenol-chloroform based extraction protocol. PCR amplification for CaStGR1 were performed with ExTaq polymerase (Takara-Fisher) using oligonucleotide primers as detailed in Supplementary Table S2. PCR products were analyzed in 1% agarose gel electrophoresis. For Sanger sequencing, PCR amplicons were purified with ExoSAP kit (Affymetrix, Santa Clara, CA, USA) to remove any excess salts carried over from PCR reactions. Amplicons were Sanger sequenced using single primers at on-campus core sequencing facilities at the University of California and the University of Vermont. Chromatogram traces from amplicon sequencing were analyzed with the Sequencher (Gene Codes Corporation, Ann Arbor, MI USA) and Geneious 2019.1 software packages. Sanger sequence traces were curated manually to identify and verify the positions of variant nucleotides in sequencing data. Variants supported by at least two independently run sequencing reaction were recorded and used for enumerating allele distribution and frequencies.

Preparation of whole genomic libraries for Illumina sequencing and data analysis of Illumina short read data were as described previously [65]. Illumina reads were mapped to the *C. arietinum* ‘CDC Frontier’ reference genome assembly [50] using BWA MEM 0.7.9a-r786. Visualization of CaStGR1 and its flanking regions was done using an instance of GBrowse loaded with gene structural annotation available from the CDC Frontier reference.

For genotyping of the CaStGR1-1 allele as a CAPS marker, PCR products were digested with Hpy-188I restriction enzyme (New England Biolabs, USA) per manufacturer’s recommended protocol. Digested PCR products were analyzed by gel electrophoresis in 1.35% agarose gels in 0.5× Tris Borate EDTA buffer stained with cybersafe reagent. Genotyping of the CaStGR1-4 allele in F2 population of wild type (yellow cotyledon) genotypes and green-cotyledon lines was conducted as a customized KASP assay (LGC Genomics, UK) using leaf tissue from greenhouse grown plants and oligos listed in Supplementary File S2.

### 4.3. Plant Growth Conditions for Physiological Assays (Experiments Listed in Table 3)

#### 4.3.1. Experiments Conducted in Glass-House (Experiment 1, 2, 3a and b)

The glass-house environment was used to evaluate crop responsiveness to soil and atmospheric drought. In Experiments 1 and 2 (Supplementary Table S4), plants were grown in 8” plastic pots filled with 5 kg of vertisol while for experiment 3a and b, plants were raised in PVC cylinders filled with 45 kg of vertisol. The experiments were set-up using completely randomized block design with treatments as separate blocks. The black soil (Vertisol) was collected from the ICRISAT farm and fertilized with DAP (di-ammonium phosphate) at the rate of 0.3 g per kg of soil in all experiments. Seeds were treated with fungicides (Thiram^®^; Sudhama Chemicals Pvt. Ltd. Gujarat, India) to avoid fungal contamination. Four seeds were sown in each pot, and a rhizobium inoculum (Strain No: IC 2002) was added to each pots to ensure adequate nodulation. Two weeks after sowing, plants were thinned to two plants per pot. Plants were maintained well-watered up to ~30 days after sowing. During the experiments duration, a data logger (Lascar Electronics Inc. Whiteparish, UK) was positioned within the plant canopy for the hourly recording of the air temperature and relative humidity (RH%) and these oscillated on average between 28–22 °C and 70–90% during the day–night cycle.

#### 4.3.2. Experiments Conducted at LeasyField (Experiment 3c)

The Lysimetric facility located at International Crops Research Institute for the Semi-Arid Tropics (ICRISAT) Patancheru in India (17°30′N; 78°16′E; altitude 549 m). It offers an experimental setup to evaluate the basic crop agronomic features, monitor the crop capacity to convert water into biomass (g of dry mass per unit of water transpired) and to measure water use patterns during the cropping season. Plants were grown in lysimeters constructed from the PVC plumbing pipes with 20 cm diameter and 1.2 m length outdoors under a rain-out shelter (ROS) (Experiment 3c). The protocol for lysimeter soil preparation & filling, spacing arrangement, growing and weighing plants were followed according to [69,70] and [60,77]. Three seeds were sown in each cylinder and watered regularly and around 15 DAS thinned to one seedling per cylinder. The experiment was planned in a complete randomized block design. One block was assigned to a well-watered treatment (WW) and two blocks to water-stressed treatment (WS). The WS treatment was imposed by cessation of watering from 25 Days after sowing (DAS). WW plants were watered every week to maintain 80% field capacity until maturity. During the experiment’s duration, the data logger was positioned within the plant canopy to record the day and night temperatures and relative humidity (RH%), which fluctuated under the natural day–night oscillations around average 31.7/15.5 °C and 40/85%.

#### 4.3.3. Experiments Conducted at LeasyScan (Experiment 4)

LeasyScan is a high throughput phenotyping platform constructed to monitor crop canopy related parameters during the vegetative phase of development with high throughput and accuracy. Details of LeasyScan technology and set-up are elaborated in [61,62,64]. For experiment 4 the crop was raised in large trays (60 × 40cm, approximately 75 kg of vertisol; i.e., “miniplots”) filled with vertisol using the recommended field management practices (20 kg·ha^−1^ of DAP and planting densities of 32 plants m^−2^). The experimental design was an Alpha lattice with 4 replications to account for spatial variability. Plants were maintained under well water conditions throughout the experiment. Canopy size related parameters (i.e., 3D-Leaf area, digital biomass and leaf area index) were continuously measured from 15-40 DAS when the plants were harvested. During the crop grown period the daily temperature and humidity oscillated in between of 11/35.8 °C and 17.2/93.2% on average as per the records of the attached weather station (Model: WxPRO™; Campbell Scientific Ltd., Shepshed, UK).

#### 4.3.4. Experiments Conducted in Field (Experiment 5)

The main crop agronomic features were measured in the field experiment that was planted in post-rainy 2017–18 season at ICRISAT field facilities. The field was solarized using a polyethene mulch during the preceding summer primarily to avoid the crop infection by *Fusarium oxysporum* f. sp, [78]. The basal dose of di-ammonium phosphate at the rate of 18kg N ha^−1^ and 20kg P ha^−1^ was applied before sowing. The field was prepared as broad bed and furrows with 1.2m wide beds flanked by 0.3m furrows. Within these beds, the plots of 4 rows of 4 m length were planted. Seeds were treated with Thiram^®^ (Sudhama Chemicals Pvt. Ltd. Gujarat, India) to avoid fungal contamination during germination. The seeds were hand sown at a depth of 2–3 cm maintaining a row-to-row distance of 30 cm and a plant to plant distance of 10 cm (i.e., 33 plants m^−2^). After sowing, furrow irrigation (60 mm) was given to ensure uniform seedling emergence. Subsequently, plants were grown under different irrigation regimes: water stress [WS; crop received only ~60 mm at the sowing], and well water [WW; crop received ~60mm at the sowing and additional ~20 mm irrigation every 20 days through perforated irrigation system]. The plots were kept weed-free by hand weeding and intensive protection was taken against pod borer (*Helicoverpa armigera*). The experiment was conducted in a randomized complete block design with three replications for each treatment (WW/WS).

### 4.4. Physiological Assays

#### 4.4.1. Experiments to Test Plant Responsiveness to Soil Drought (Experiment 1b and 1c)

The main aim of “dry-down” experiments is to assess the capacity of genotypes to restrict the transpiration upon declining soil moisture, which could be a crucial adaptive trait for plants in particular water-limited environments. To test the transpiration restriction capacity of selected genotypes, these were organized in two experimental blocks; well-watered (WW) and water-stressed (WS) conditions. The day before the dry-down was initiated all pots were abundantly watered and the soil was allowed to drain overnight. The following day the soil surface of the pots were covered with plastic sheets, and then a uniform 2 cm layer of plastic beads to prevent soil evaporation. The pots were then weighed and this initial pot weight was considered as the soil-saturation level (field capacity) of the individual pots. Pot weight was recorded daily at the same time of day. Based on the daily weight loss, the well-watered plants were maintained at approximately 80% of the saturated weight (80% of the field capacity). For the WS treatment, the water available to the plant was gradually decreased by allowing a maximum daily water loss of 70g. The transpiration weight loss above 70g was compensated by adding an excess amount of transpired water to each pot. The experiment was terminated when transpiration of all WS plants was below 10% of their WW treated counterparts. After termination, the above-ground biomass of the plants was harvested, organs separated, and oven-dried at 60 °C for a minimum of 3 days. The traits assessed are detailed in Supplementary Table S4.

Additionally, during the dry-down experiments (in Experiment 1b and 1c), 30 mg leaf tissue (leaflets from the first fully developed leaf from the top of the main stem) from each replicate (i.e., in WW and WS) were collected twice WW and severe water stress (~0.25 NTR). Collected tissues were frozen by liquid nitrogen and conserved for later estimation of pigments (i.e., Chlorophylls and Carotenoids, see below). (http://gems.icrisat.org/allinstruments/controlled-imposition-of-water-stress/; methodology also used in e.g., [79,80,81])

#### 4.4.2. Experiments to Test Plant Responsiveness to Atmospheric Drought (Experiment 2a,b,c)

While “dry-down” experiments (above, experiment 1b and c) were conducted to evaluate plant responsiveness to drying soil, complementary “transpiration responsiveness” experiments were designed to characterize the genotypic ability to limit transpiration upon drying atmosphere [increasing vapour pressure deficit (VPD)]. For this, the plants were evaluated during vegetative growth stage under well-watered conditions. Around 30-day-old plants grown in pots were watered to ~90% field capacity and soil evaporation minimized by applying the plastic sheets and beads similarly as in the regulated dry down experiment (above). Initially, the plant transpiration was evaluated outdoors during the cloud-less clear days in the natural circadian cycle or in the growth chambers (Conviron-PGW36 model, Controlled Environments Limited, Winnipeg Manitoba, Canada: see more details in http://www.conviron.com/sites/default/files/PGW36%20Data%20Sheet_1.pdf). In these experiments, temperature and humidity sensors were mounted at canopy level to record the actual conditions experienced by the crop canopy in 5 min intervals. In the outdoors conditions, plants were weighted in hourly intervals using 0.01 g precision scales (KERN 24100, Kern & Sohn GmbH, Balingen, Germany). Consequently, for the controlled environment testing, the same pots were placed into the growth chamber for one day to acclimate with the day/night temperature (°C) and relative humidity (RH%) of 32/26 °C and 60/80% respectively. Plants were then exposed to an increasing ladder of VPD ranging from 0.9 to 4.1 kPa by increasing temperature and decreasing RH% (80–30%) at hourly intervals for 8 h. Plant transpiration was also assessed hourly by swift weighing in between of the VPD transitioning regimes. At the end of the experiments, plants were harvested and leaf area (LA) was measured with a leaf area meter (LI-3100C area meter, LI-COR^®^Biosciences, Lincoln, NE, USA). Consequently, the plant transpiration rate was expressed as TR = T/LA [g of water transpired per unit of LA per hour] and regressed upon VPD during the particular time interval. In both germplasm and ILs (Experiment 2a and 2c), the specific leaf weight (SLW) was estimated as leaf dry weight (g)/leaf area (cm^−2^).

(http://gems.icrisat.org/allinstruments/transpiration-response-to-increasing-vpd/; methodology also used in e.g., [61,62,79,80])

#### 4.4.3. Experiments to Test Plant Baseline Agronomic Features and Water-Use Related Traits in Lysimetric Facility (Experiment 3a,b,c)

The unique lysimetric set-up allows estimating the plant water productivity while having access to relevant agronomic traits. The cylinders were covered with plastic sheets and beads similarly as in assay #1 and 2 and the water use monitoring started ~25 DAS. From this onwards, the cylinders were weighed weekly by lifting them with a block chained pulley using S-type load cell (Mettler-Toledo, CSE 100, Geneva, Switzerland) until crop maturity. The WW block of experimental plants was retained at 80% of field capacity. Under the WS treatment, the declining soil moisture was only monitored but not regulated, which contrasts with the regulated dry-down protocol used in the pot culture (see above #1). During the plant growth flowering dates were recorded for each plant. At the end of the experiment, plants were harvested, the crop residuals dried at 60 °C in an oven during minimum 72 h and the above ground biomass, grain and vegetative dry biomass were weighed (KERN 3600 g; 0.01 g precision balance, Kern & Sohn GmbH, Balingen, Germany). Plant transpiration was calculated from consecutive cylinder weight differences and water additions. Transpiration efficiency (TE; [gram of biomass per kilogram of water transpired; g/kg^−1^]) and water use efficiency (WUE, [gram of seed weight per kilogram of water transpired; g/kg^−1^]) was then calculated as the ratio of the total/grain dry biomass per unit of water transpired. Lastly, Harvest Index (HI) was calculated as the ratio of total dry grain biomass per the total dry weight of remaining above-ground biomass. (http://gems.icrisat.org/allinstruments/lysimetric-assessments/, methodology also used in e.g., [60,69,70,82,83]).

#### 4.4.4. Experiments to Assess Plant Canopy at LeasyScan (Experiment 4)

The LeasyScan platform has been used to monitor traits indicating crop canopy traits related to “vigor”. This is enabled by the optical system (PlantEye^®^; www.phenospex.com), which captures the dynamics of canopy growth during the crop vegetative growth-phase with high throughput and accuracy. We measured 3D-Leaf area (3D-L; canopy size reconstructed from 3D point-cloud distribution [mm^3^]), projected leaf area (PL; canopy ground coverage [mm^2^]) and plant height (PH; estimated from 3D point-cloud as height encompassing 95% of recorded points of given point-cloud) during 15-30 DAS (http://gems.icrisat.org/leasyscan/) methodology also used in e.g., [4,61,64]).

#### 4.4.5. Agronomic Evaluation of ILs in Field Settings (Experiment 5)

Agronomic traits of selected stay-green introgression lines and their recurrent parents were evaluated using the precision field facility under optimal water input (WW) and under severe water shortage WS. Under both treatments, in each plot we monitored the phenology parameters (date to first flower, 50% flowering and 80% of the dried pods was recorded as maturity). At maturity, shoots were harvested plot wise and kept for drying at 60 °C for minimum of 3 days. Organs were separated, dry weights recorded and expressed in grams per meter square (g m^−2^). 100 seed number was counted by seed counter (Data Count S60 seed Counter, Data technologies, Israel; http://www.data technologies.com/data_count_s_60_seed_counter.html), weighed and based on these the total seed number per square meter was calculated.

Harvest index was calculated:                    HI = (Seed weight/total shoot biomass weight) × 100 [%].(1)

### 4.5. Chlorophyll and Carotenoid Estimation in Leaves and Seeds (Measured in Experiment 1 and 3)

Photosynthetic pigment contents (chlorophyll a, chlorophyll b and total carotenoids) were assessed in the leaf tissues across various stages of plant exposure to declining soil content in lysimeters (un-regulated dry-down; Experiment 3) and in pot cultures (regulated dry-down; Experiment 1b and 1c). The grain pigments were assessed only in the experiments conducted at lysimetric experiments (Experiment 3a,b,c).

In Experiment 3c the leaf tissue samples were collected from each plant from the glasshouse lysimetric experiment. Chlorophyll a and b, as well as Carotenoids, were estimated from the samples using dimethyl sulfoxide (DMSO) method [84]. We standardized that around 18 mg of fresh leaf tissue/30mg of dry-seed powder extracted in a 5mL of DMSO resulted in suitable optical density (OD) between 0.3–0.9. The test-tubes with the exact weighted tissue and DMSO were placed in ~65 °C hot water bath and left for cca 3 h until the tissue became translucent ensuring all pigments were extracted into to the DMSO. The OD of extract was assessed spectrophotometrically (Shimadzu UV-2401 PC UV-Visible Spectrometer; Shimadzu Scientific Instruments) at 665.1 (Chlorophyll A), 649.1 (Chlorophyll B) and 480 (Total Carotenoids) and the contents were calculated as per [84].

The grain material from Experiment 3b was used to separate the main carotenoids using the High Performance Liquid Chromatography (HPLC) system. For this, the extraction of carotenoids was done according to the method of [85] with some modifications. Briefly, about 0.1 g of chickpea sample was weighed and placed in a screw-capped glass tube (~15 mL tube) and 1 mL ethanol containing 0.1% butylated hydroxytoluene (BHT) added to the solution. The mixture was saponified by adding 200 µL of 20% Potassium hydroxide (KOH) and mixed by vortexing. Extraction was completed by adding 1.5 mL hexane to the saponified solution, vortexed for 20 s and centrifuged at 2500 rpm for 5 min. Using a glass pipette, the upper hexane layer containing carotenoids was carefully removed and transferred to a new glass tube. Extraction was repeated 2 more times. The combined hexane extracts were then dried down under a stream of nitrogen gas. Purified β-apo-8′-carotenal was used (absorbance ~ 0.8; 100 µL) was used as an internal standard. The dried extract was reconstituted in 100 µL of 50:50 (*v/v*) methanol:dichloroethane and 10 µL of the sample injected into the HPLC system (duplicate injections per sample).

Chromatographic separation of carotenoids was carried out using the Ultra-Fast Prominence Liquid chromatography (Shimadzu, Kyoto, Japan) equipped with a SIL-20ac-xr Prominence auto-sampler, a DGU-20A5 Prominence degasser, a CTO-20AC column oven and an SPD-M20A Diode Array Detector (DAD). Separation of carotenoids was achieved at 25 °C on a C30 YMC carotenoid column (250 × 4.6 mm, i.d., 5 µm particle size, Waters, Ireland) on a gradient method with 95% Methanol as solvent A and 100% MTBE as solvent B. Identification of the carotenoids was based on the standards, their retention times and by comparing the absorption spectra with those in the literature. Quantification of the carotenoids were extrapolated from standard curves prepared from authentic standards after correcting for extraction efficiency based on the recovery of the internal standard. The processing of all chromatograms was done using Shimadzu LC Lab-Solutions software (also used in [26,84,85]).

### 4.6. Statistical Analysis

In the experiments 1b, 1c, 2a, 2c, 3a, 3b, 3c, 4 and 5, the differences between investigated genotypes were evaluated by simple/multiple-way ANOVA followed by the Tukey–Kramer test to evaluate the significance of genotypic differences (Statistical program package CoStat version 6.204 (Cohort Software, Monterey, CA, USA). The line graph (Experiment 2a, 4), bar graph (1b, 1c, 2a, 2c, 3a, 3b, 3c, 4 and 5) and simple linear regressions were fitted using Microsoft Excel 2013 (Microsoft Corp., Redmond, WA, USA). For treatment of temporal data from experiments 1b, 1c and experiments 2 a,c-i.e., transpiration response to atmospheric (Experiment 2a and 2c) and soil drought (Experiment 1b and 1c) we used methodologies described in [69,70,80,86,87]; specifically, a nonlinear regression analysis was done using GraphPad Prism version 6 (GraphPad Software Inc., San Diego, CA, USA), and Genstat 14.0 (VSN International Ltd., Hemel Hempstead, UK).

## 5. Conclusions

Chickpea production suffers greatly due to its cultivation predominantly as a rain-fed crop, particularly across developing countries. Significant progress has been made from crop agronomic practices and breeding to address the yield gap to ensure appropriate caloric intake of populations inhabiting these areas. Although caloric intake is slowly increasing, human nutrient deficiencies prevail in the same regions and remain largely unaddressed. Therefore, in this paper we tested the suitability of stay-green chickpea for cultivation in semiarid tropical regions, which as a genetic biofortification technology may help to reduce widespread vitamin-A deficiency while maintaining the levels of agronomic production. We tested a range of plant material with the stay-green character which was expressed as an extended maintenance of chlorophylls and carotenoids in dry seeds and leaves. We found this particular phenotype was controlled by variation in a single gene, CaStGR1, an ortholog of Mendel’s I locus of garden pea, which occurred in 5 different allelic variants in the tested material. We also showed that across a range of environmental conditions the stay-green allelic variants were very likely neither influencing the mechanisms linked to drought stress adaptations nor negatively influencing important agronomic traits. Our evidence that the green-seeded CaStGR1 variants contain multiple-fold higher levels of the phytonutrients lutein, and provitamin A (beta-carotene) when compared to the more common yellow cotyledon chickpea indicate a higher nutritional value of the green cotyledon type. Further investigations of the bioavailability of vitamin A, multilocation trials for yield stability, and acceptability of the stay-green chickpea products in production regions by producers and consumers are warranted in order to establish the efficacy of genetic biofortification with stay-green chickpea for improving human nutrition and health.

## Figures and Tables

**Table 1:** Summary of nucleotide variants identified in CaStGR1 among chickpea germplasm. The color of cotyledons, designated allele names for the variants, the nature of molecular lesions found in each allele, and their frequencies among germplasm studied are listed.

Cotyledon Color	Allele	Nucleotide Variation in CaStGR1	Number of Genotypes
green	CaStGR1-1	1-bp “g“ del in exon 1	7
green	CaStGR1-2	8-bp “ctaggttg“ deletion in exon 2	5
green	CaStGR1-3	1-bp “c“ deletion in exon 2	10
green	CaStGR1-4	1-bp “g“ del in exon 4	6
green	CaStGR1-5	entire gene deleted	11
Yellow/Tan	CaStGR1 WT	“Wild Type”	6

**Table 2:** Regression analysis of transpiration response to soil drying of green cotyledon trait donor genotype ICC 16340, recurrent yellow cotyledon elite cultivars KAK-2 and JGK1 and backcross introgression lines of the green cotyledon trait in these elite cultivar backgrounds.

Genotypes	NTR-FTSW Thresholds and Std. Error	Slope 1 and Std. Error	Slope 2 and Std. Error
ICC 16340_Stg-D-P	0.58 ± 0.02	1.92 ± 0.08	−0.59 ± 0.23
KAK2_R-P	0.54 ± 0.03	1.72 ± 0.05	0.20 ± 0.26
JGK 1_R-P	0.54 ± 0.03	1.88 ± 0.06	−0.12 ± 0.24
ICCX-060119-107 (KAK2)	0.54 ± 0.03	1.85 ± 0.08	−0.10 ± 0.22
ICCX-060119-113 (KAK2)	0.48 ± 0.03	1.98 ± 0.09	0.06 ± 0.17
ICCX-060119-116 (KAK2)	0.58 ± 0.03	1.64 ± 0.07	0.01 ± 0.19
ICCX-060119-123 (KAK2)	0.62 ± 0.05	1.69 ± 0.09	−0.32 ± 0.53
ICCX-060121-125 (JGK1)	0.51 ± 0.02	1.87 ± 0.06	−0.01 ± 0.17
ICCX-060121-128 (JGK1)	0.60 ± 0.03	1.63 ± 0.06	0.05 ± 0.28
ICCX-060121-129 (JGK1)	0.55 ± 0.03	1.72 ± 0.07	0.14 ± 0.18

**Table 3:** Regression analysis of transpiration response to VPD in outdoor and growth chamber of green cotyledon trait donor genotype ICC 16340, recurrent yellow cotyledon elite cultivars KAK-2 and JGK1 and backcross introgression lines of the green cotyledon trait in these elite cultivar backgrounds.

Genotypes	TR Response to VPD at Outdoor		TR Response to VPD at Growth Chamber		
**KAK-2 Background**	**Mean TR & SE** **LSD (0.01) = 0.09**	**Slope at high VPD & SE** **LSD (0.01) = 0.59**	**Mean TR & SE** **LSD (0.001) = 0.35**	**Slope & SE** **LSD (0.01) = 0.04**	**R^2^**
ICC 16340_Stay-green_Donor Parent	1.31 ± 0.04a	5.48 ± 0.18a	2.02 ± 0.06a	0.52 ± 0.06a	0.94
KAK2_Recurrent parent	0.95 ± 0.02b	4.18 ± 0.08b	1.45 ± 0.06b	0.41 ± 0.04b	0.94
ICCX-060119-107 (KAK2 Background)	0.98 ± 0.04b	4.16 ± 0.18b	1.37 ± 0.04b	0.43 ± 0.04b	0.95
ICCX-060119-113 (KAK2 Background)	1.04 ± 0.02b	4.23 ± 0.06b	1.49 ± 0.06b	0.46 ± 0.04b	0.95
ICCX-060119-116 (KAK2 Background)	0.94 ± 0.04b	4.19 ± 0.20b	1.45 ± 0.08b	0.47 ± 0.05b	0.94
ICCX-060119-123 (KAK2 Background)	1.03 ± 0.02b	4.16 ± 0.16b	1.63 ± 0.04b	0.40 ± 0.05b	0.91
**JGK-1 Background**	**Mean TR & SE** **LSD (0.001) = 0.12**	**Slope at high VPD & SE** **LSD (0.01) = 0.46**	**Mean TR & SE** **LSD (0.01) = 0.25**	**Slope & SE** **LSD (0.01) = 0.033**	**R^2^**
ICC 16340_Stay-green_Donor Parent	1.31 ± 0.04a	5.48 ± 0.18a	2.02 ± 0.06a	0.52 ± 0.06a	0.94
JGK 1_Recurrent parent	1.14 ± 0.04b	4.77 ± 0.16b	1.70 ± 0.06b	0.45 ± 0.04b	0.95
ICCX-060121-125 (JGK1 Background)	0.99 ± 0.03bc	4.09 ± 0.01c	1.53 ± 0.06b	0.42 ± 0.04b	0.95
ICCX-060121-128 (JGK1 Background)	0.90 ± 0.03c	3.82 ± 0.08c	1.52 ± 0.08b	0.38 ± 0.04b	0.94
ICCX-060121-129 (JGK1 Background)	0.90 ± 0.01c	3.78 ± 0.05c	1.52 ± 0.05b	0.37 ± 0.04b	0.94

## Supplementary Materials

Supplementary materials can be found at https://www.mdpi.com/1422-0067/20/22/5562/s1.
