# Supplementary figures and images for: Functional Dissection of the Chickpea (Cicer arietinum L.) Stay-Green Phenotype Associated with Molecular Variation at an Ortholog of Mendel’s I Gene for Cotyledon Color: Implications for Crop Production and Carotenoid Biofortification
Source: Int J Mol Sci. 2019 Nov 7;20(22):5562. doi: 10.3390/ijms20225562 (PMC6888616; doi:10.3390/ijms20225562)

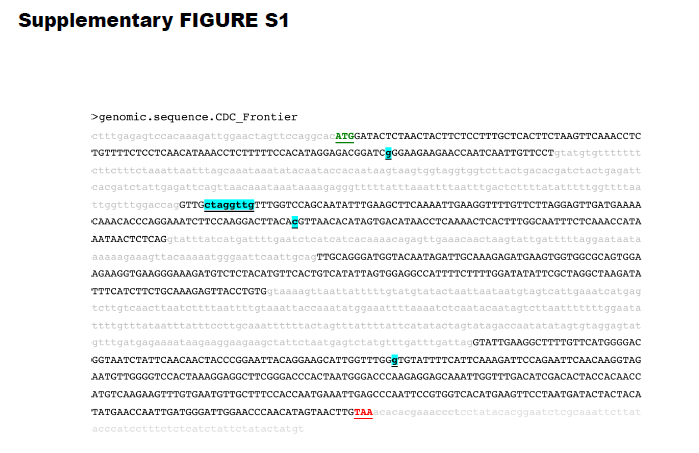


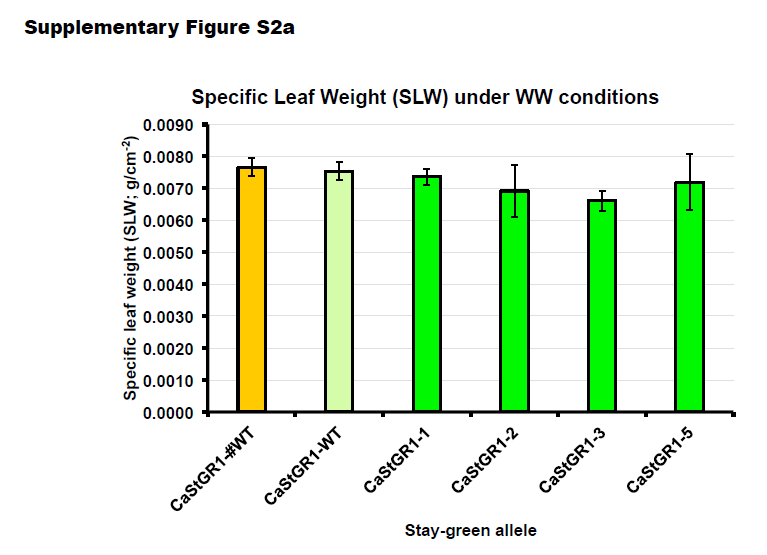


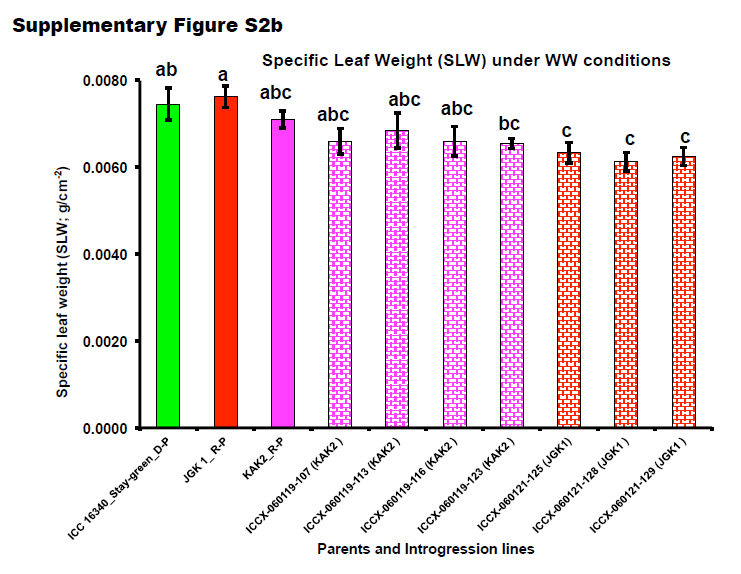


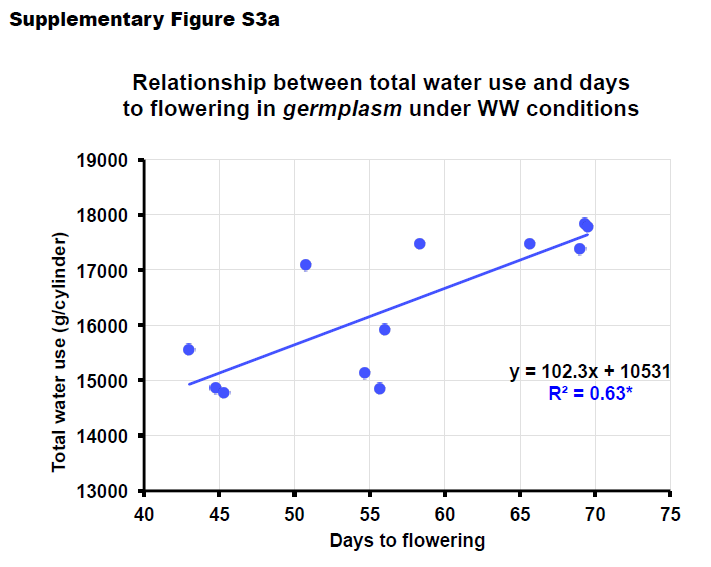


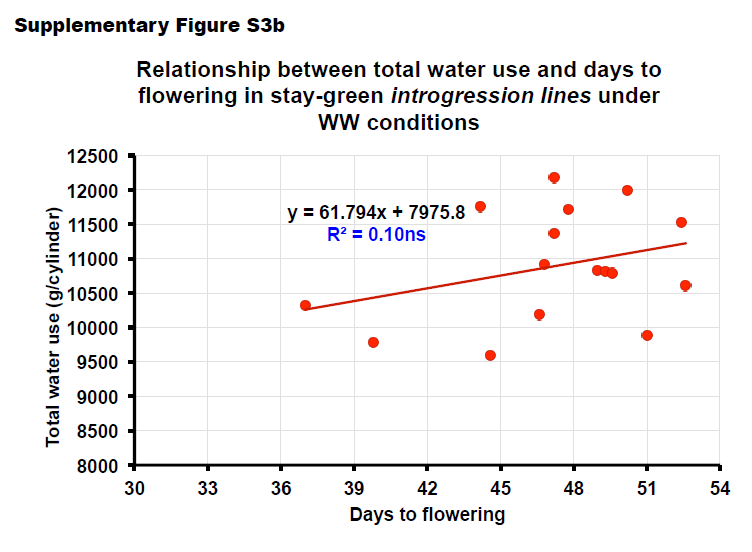


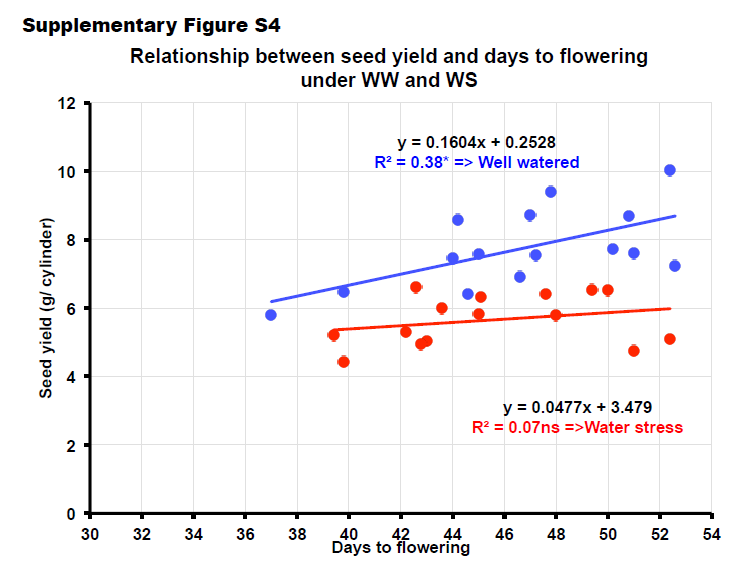


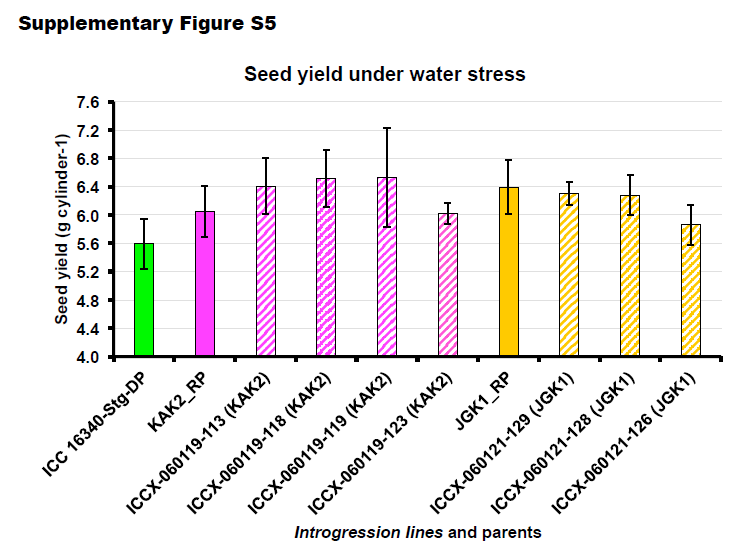


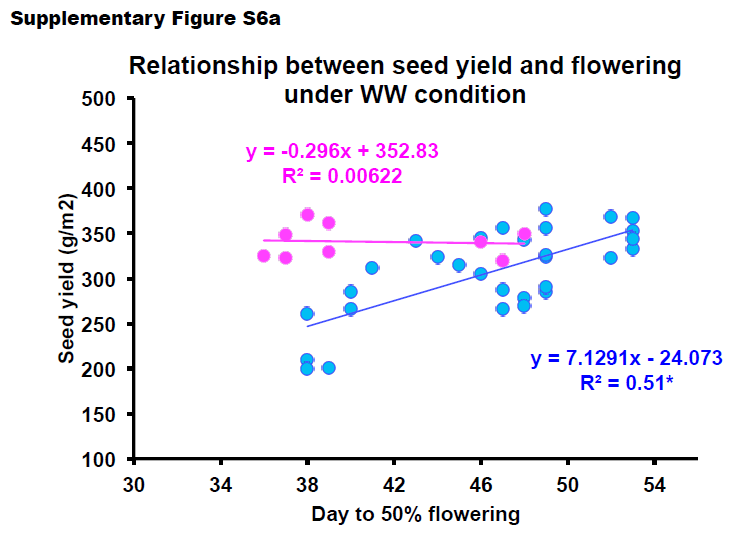


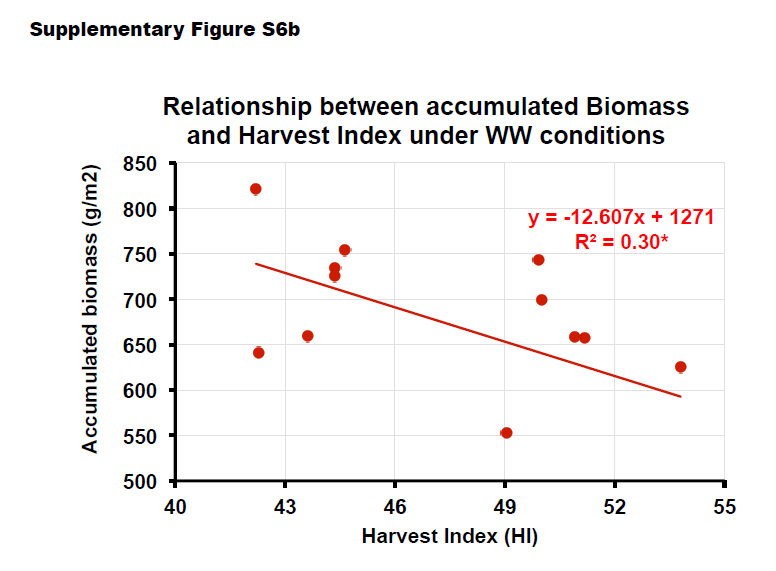


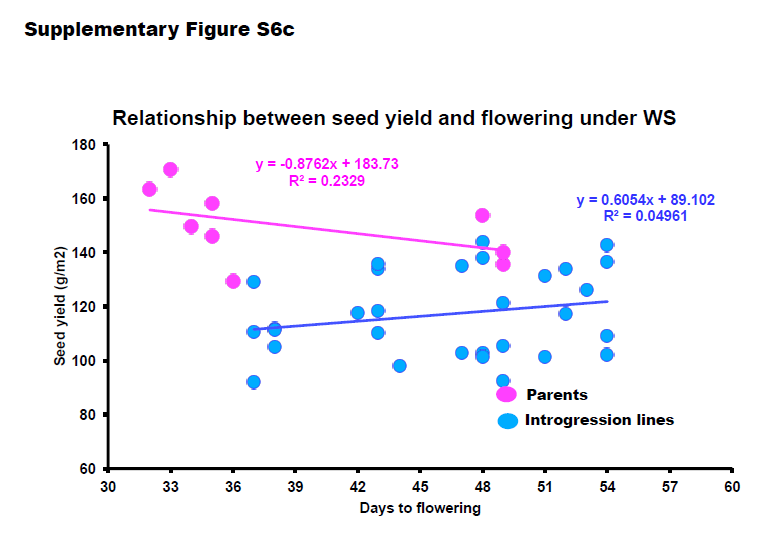


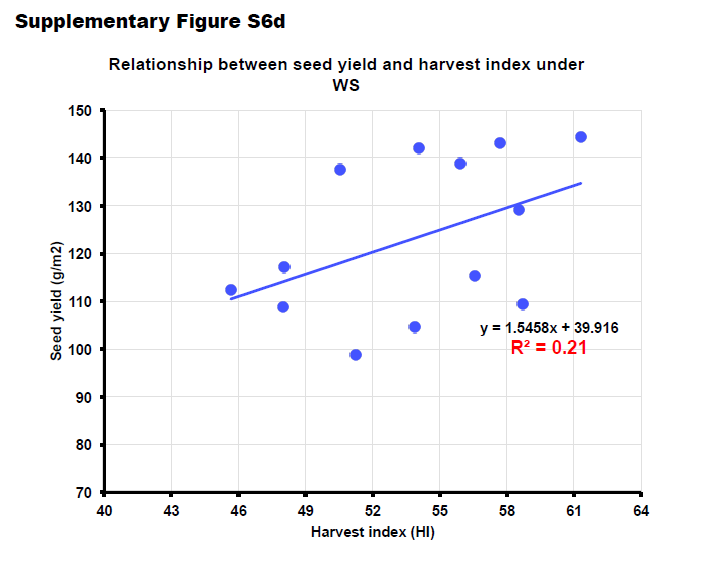


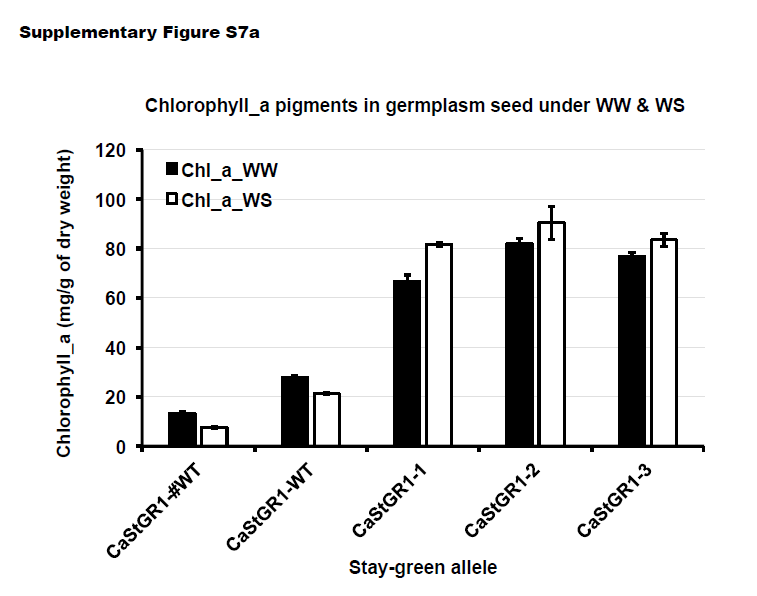


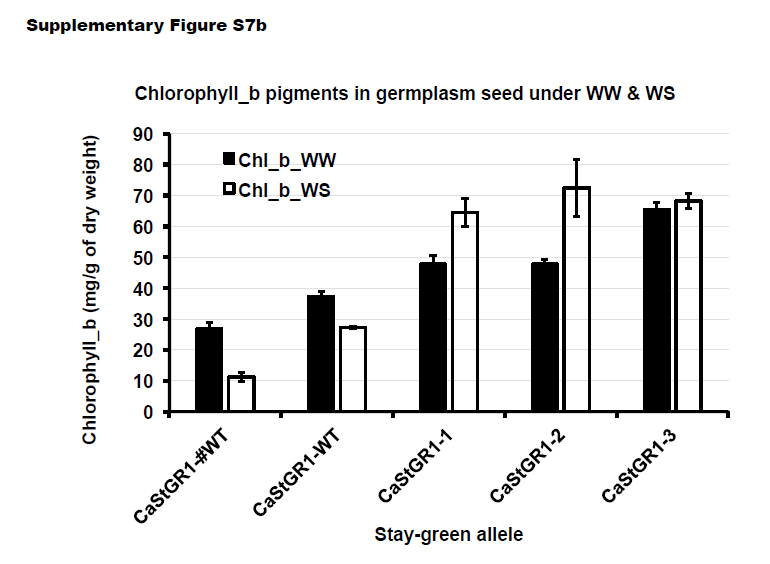


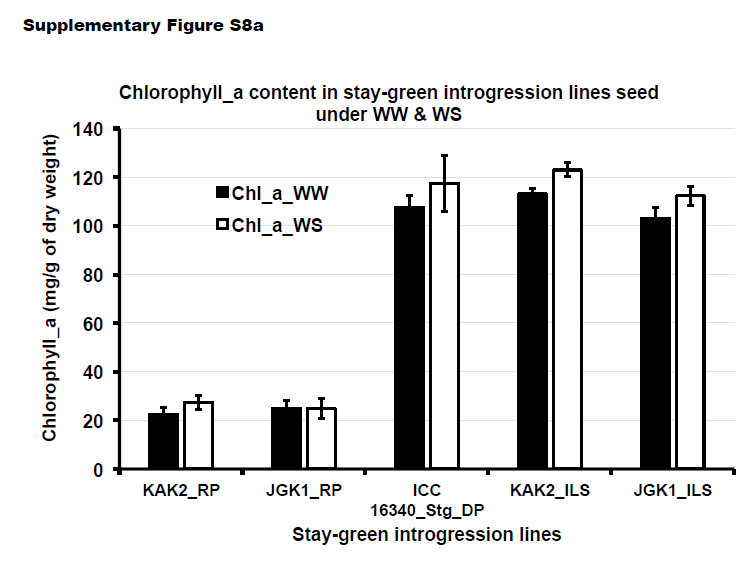


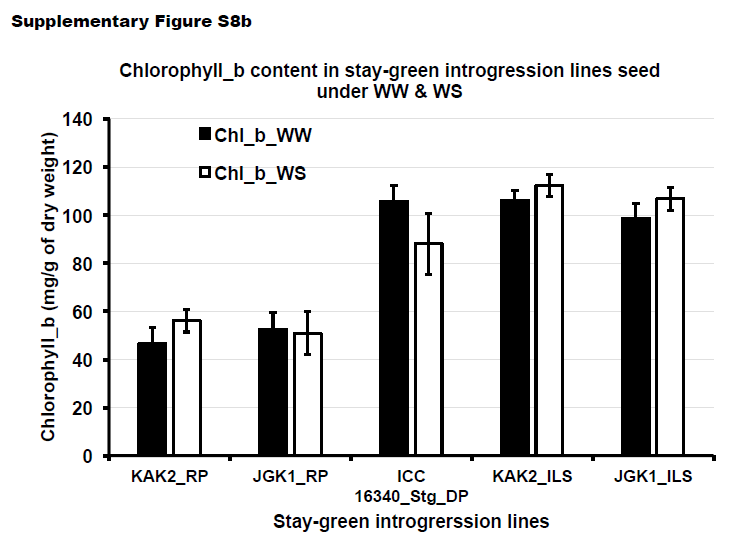

Supplement: Supplementary file 1 [file ijms-20-05562-s001.zip › ijms-632989 sup/ijms-632989 sup fig.docx]
